# Water‐limited environments affect the association between functional diversity and forest productivity

**DOI:** 10.1002/ece3.10406

**Published:** 2023-08-08

**Authors:** Roel Lammerant, Angelo Rita, Marco Borghetti, Robert Muscarella

**Affiliations:** ^1^ Department of Ecology & Genetics Uppsala University Uppsala Sweden; ^2^ Dipartimento di Agraria Università degli Studi di Napoli Federico II Portici (Napoli) Italy; ^3^ Scuola di Scienze Agrarie, Forestali, Alimentari ed Ambientali Università degli Studi della Basilicata Potenza Italy; ^4^ Present address: Tvärminne Zoological Station University of Helsinki Hanko Finland

**Keywords:** diversity–productivity relationship, forest productivity, functional diversity, National Forest Inventory, plant traits, structural equation modeling

## Abstract

The link between biodiversity and ecosystem function can depend on environmental conditions. This contingency can impede our ability to predict how biodiversity‐ecosystem function (BEF) relationships will respond to future environmental change, causing a clear need to explore the processes underlying shifts in BEF relationships across large spatial scales and broad environmental gradients. We compiled a dataset on five functional traits (maximum height, wood density, specific leaf area [SLA], seed size, and xylem vulnerability to embolism [P_50_]), covering 78%–90% of the tree species in the National Forest Inventory from Italy, to test (i) how a water limitation gradient shapes the functional composition and diversity of forests, (ii) how functional composition and diversity of trees relate to forest annual increment via mass ratio and complementarity effects, and (iii) how the relationship between functional diversity and annual increment varies between Mediterranean and temperate climate regions. Functional composition varied with water limitation; tree communities tended to have more conservative traits in sites with higher levels of water limitation. The response of functional diversity differed among traits and climatic regions but among temperate forest plots, we found a consistent increase of functional diversity with water limitation. Tree diversity was positively associated with annual increment of Italian forests through a combination of mass ratio and niche complementarity effects, but the relative importance of these effects depended on the trait and range of climate considered. Specifically, niche complementarity effects were more strongly associated with annual increment in the Mediterranean compared to temperate forests. *Synthesis*: Overall, our results suggest that biodiversity mediates forest annual increment under water‐limited conditions by promoting beneficial interactions between species and complementarity in resource use. Our work highlights the importance of conserving functional diversity for future forest management to maintain forest annual increment under the expected increase in intensity and frequency of drought.

## Graphical abstract

Biodiversity and ecosystem function relationships can depend on abiotic context. We used National Forest Inventory data from Italy to test the relationship between functional diversity and forest productivity. Our results suggest that biodiversity plays an important role in driving forest annual volume increment under water‐limited conditions by promoting beneficial interactions between species and complementarity in resource use.

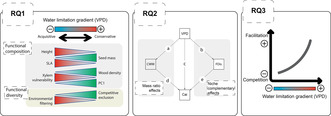

## 1 INTRODUCTION

A compelling amount of empirical evidence has demonstrated that biodiversity can affect ecosystem functioning, with many studies supporting positive biodiversity‐ecosystem function (BEF) relationships (Cardinale et al., 2011; Chapin et al., 1997; Loreau et al., 2001; Ratcliffe et al., 2016; Wright et al., 2021). The importance of BEF effects can be context‐dependent, changes in environmental conditions may alter the strength and shape of these relationships (Pretzsch et al., 2013; Ratcliffe et al., 2016; Wright et al., 2021). This impedes our ability to predict how BEF relationships will respond to rapid global change. As such, there is a clear need to explore the processes underlying shifts in BEF relationships across large spatial scales and broad environmental gradients (Baert et al., 2018; Fei et al., 2018; Gonzalez et al., 2020; Grossiord, 2020; Hisano et al., 2018; Paquette et al., 2018; Ratcliffe et al., 2017).

Characterizing communities in terms of their functional composition and diversity can help reveal the factors shaping patterns of biodiversity and community structure, as well as the influence of biological communities on ecosystem functioning (Augusto & Boča, 2022; Bonilla‐Valencia et al., 2022; Díaz & Cabido, 2001; Laureto et al., 2015; Petchey et al., 2004). Numerous studies have shown that considering the functional traits of organisms can improve our understanding of how biodiversity affects ecosystem‐scale processes by providing a more physiological basis for the ecological function of species in communities (Cadotte et al., 2011; Díaz & Cabido, 2001; Song et al., 2014; Yan et al., 2023). For example, Ayma‐Romay et al. (2021) showed that the inclusion of traits, which capture key variation in plant life‐history strategies, representing a trade‐off between conservative and acquisitive resource strategies, can help explore the mechanisms that underlie BEF relationships.

Two main mechanisms are generally considered to underlie BEF relationships: niche complementarity (or resource partitioning) and mass ratio (or dominance) effects (Loreau et al., 2001). Niche complementarity can be reflected by the functional diversity of a community; a greater variety of trait values in a given community can lead to higher rates of ecosystem function due to more efficient exploitation of resources (Sonkoly et al., 2019). Mass ratio effects refer to the composition of a community; the trait values of the most abundant species in a community are expected to have the largest effect on the relationship between diversity and ecosystem function (Ali, 2015; Loreau et al., 2001; Sonkoly et al., 2019). Previous studies have produced contrasting results regarding the relative importance of these two mechanisms to explain BEF relationships (Ammer, 2019; Grossman et al., 2018; Wright et al., 2021). However some common patterns have emerged; specifically, environmental stress (e.g., water limitation) appears to be a major factor governing the relative importance of niche complementarity versus mass ratio effects. For example, Richardson et al. (2012) showed that the relative importance of complementarity and mass ratio effects varied inversely with a latitudinal gradient in grasslands, with a greater relative importance of complementarity effects in more water‐limited environments.

The stress gradient hypothesis (SGH; Bertness & Callaway, 1994) provides a framework for predicting how environmental conditions may mediate BEF relationships. Specifically, the SGH predicts that the frequency of facilitative and competitive interactions will vary inversely across stress gradients (Bertness & Callaway, 1994; Maestre et al., 2009). In relatively benign conditions (e.g., mesic forests), competitively dominant species will drive rates of ecosystem function via mass ratio effects. Under higher abiotic stress (e.g., xeric forests), in contrast, facilitation among species will lead to more efficient use of available resources at the community level and outweigh the negative competitive impacts of neighbors. As a result, the role of competitive interactions becomes relatively weak compared to niche complementarity effects via resource partitioning (Richardson et al., 2012; Schmitt et al., 2020).

To better understand how the processes influencing BEF relationships change across broad environmental gradients, we used a functional trait‐based approach over an extensive spatial scale (i.e., Italy) with a strong climate gradient. We used a National Forest Inventory dataset to examine the mechanisms by which tree diversity influences annual increment and how the relative importance of complementarity and mass ratio effects changes along a broad gradient of water limitation. We addressed the following questions:

### 1.1 How does the functional composition and diversity of forests vary with respect to abiotic gradients throughout Italy?

**FIGURE 1:**
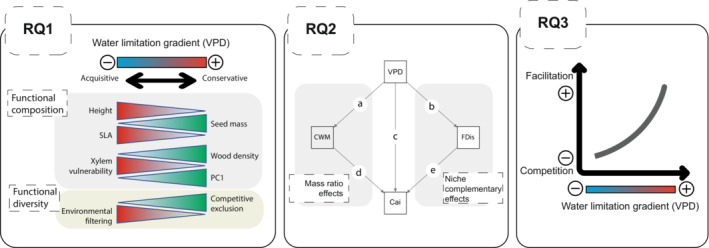
Illustration of the main research questions tested in this work. RQ1: Hypothesized response of functional composition and functional diversity against a water limitation gradient. Green triangles display increasing values, red triangles display decreasing values under increased water limitation. RQ2: Theoretical model of climate (VPD) and community functional properties (CWM and FDis) on annual volume increment (Cai). Solid arrows indicate predicted causal relationships among variables and lowercase letters are path estimates. RQ3: Stress gradient hypothesis provides a framework for understanding the mechanisms by which biodiversity influences annual volume increment, predicting that the frequency of facilitative and competitive interactions will vary inversely across abiotic stress gradients (i.e., water limitation gradient).

We expect community‐weighted mean (CWM) trait values to vary with respect to abiotic conditions in a way that reflects selection across environmental gradients. Specifically, we expect more resource‐conservative traits under increased water limitation (Figure 1, Table 1). Additionally, in more water‐limited environments, we expect lower functional diversity as a result of selection for a narrow range of more conservative growth strategies (Wieczynski et al., 2019; Figure 1). On the contrary, if competitive exclusion is the main driver behind functional diversity under favorable environmental conditions, we expect to see lower functional diversity under favorable environments in comparison with water‐limited environments (Figure 1; Levine & HilleRisLambers, 2010; Mayfield & Levine, 2010; Mensah et al., 2018).

**TABLE 1:** Hypotheses about how community‐weighted mean of traits are predicted to respond to water limitation.

Community‐weighted mean (CWM)	Units	Predicted response to water limitation	Rationale	References
Specific leaf area (SLA)	mm^2^/mg	–	Low SLA values relate to high leaf density, with tightly packed cells with thicker walls and low air spaces, which tends to increase tolerance toward water limitation	Niinemets (2001), Poorter et al. (2009) and Costa‐Saura et al. (2017, 2019)
Stem density	g cm^−3^	+	Dense wood tends to be correlated with a lower risk of drought‐induced cavitation	Hacke et al. (2001), Markesteijn, Poorter, Bongers, et al. (2011), Markesteijn, Poorter, Paz, et al. (2011), Costa‐Saura et al. (2019) and Pinho et al. (2021)
Height	m	−	Lower height tends to reduce the risk of drought‐induced cavitation	Nunes et al. (2017), Costa‐Saura et al. (2019) and Pinho et al. (2021)
Seed mass	mg	+	Larger seed mass relates to larger initial energy reserves, which allows seedlings to produce more extensive root systems to obtain water and to better tolerate drought	Metz et al. (2010), Volis and Bohrer (2013) and Costa‐Saura et al. (2019)
Xylem vulnerability (P50)	MPa	–	Lower xylem vulnerability relate to lower water potential at which species loses 50% of xylem hydraulic conductivity due to cavitation, which tends to increase tolerance toward water limitations	Choat et al. (2012) and Costa‐Saura et al. (2016, 2019)

### 1.2 How does functional composition and diversity of forests relate to annual increment?

We expect a positive relationship between tree diversity and annual increment, driven by a combination of niche complementarity and mass ratio effects (Figure 1; Ali, 2015; Loreau et al., 2001; Sonkoly et al., 2019). More specifically, we expect niche complementarity (indicated by functional dispersion) to be positively associated with site annual increment, whereas we expect the direction of mass ratio effects (indicated by CWM values) to vary with different traits (Conti & Díaz, 2013; Finegan et al., 2015; Mensah et al., 2018). In general, we hypothesize that the dominance (i.e., mass ratio effects) of species with more acquisitive functional traits (e.g., higher values of height, SLA, and P_50_, lower values of wood density, and seed mass) to be positively associated with site annual increment.

### 1.3 Is functional diversity more strongly related to annual increment in the Mediterranean or temperate climate region?

In general, forests in the Mediterranean climate region are exposed to higher levels of water limitation compared to forests in the temperate climate region. Based on the SGH, we predict a weaker relationship between functional diversity and annual increment across forests in the temperate climate if competitive interactions (i.e., mass ratio effects) are more common with lower levels of water limitation (Paquette & Messier, 2011; Rita & Borghetti, 2019; Wang et al., 2019). In contrast, we predict a stronger relationship between functional diversity and annual increment across forests in the Mediterranean climate if niche complementarity effects (i.e., resource partitioning) among plant species becomes more prevalent with higher levels of water limitation (Figure 1; Paquette & Messier, 2011; Rita & Borghetti, 2019; Wang et al., 2019).

## 2 METHODS

### 2.1 Study area and forest inventory data

The study area extends throughout Italy (35°29′–47°04′ N, 6°37′–18°31′ E, Figure S1), covering a highly variable climate gradient that ranges from Mediterranean to temperate climatic regions. More than one‐third of the country's 30 million hectares of land area is covered in forests and other woodlands (Gasparini & Tabacchi, 2011; Gasparini et al., 2022). Oak‐, beech‐, and chestnut‐dominated forests are the most common forest types, each representing over 10% of the forested land area. Spruce‐dominated woodlands are the most extensive type of coniferous forest, representing about 6% of forested land area of Italy. Because of topoclimate heterogeneity, geological history, and human influence, forests are highly diversified at a national scale in terms of tree composition, structure, and biodiversity.

We used data from the 2005 Italian National Forest Inventory (INFC; available at https://www.inventarioforestale.org/) that provides georeferenced locations (±1 km) of plots (530 m^2^) with information on species composition, abundance, and annual volume increment (m^3^ ha^−1^ year^−1^) estimated by the procedure described in Gasparini et al. (2017), which in this work we refer to as current annual increment (Cai). The full INFC dataset consists of 7272 plots distributed throughout Italy, containing >230,000 individual trees (Gasparini & Tabacchi, 2011; Tabacchi et al., 2011). For this study, we excluded plots identified as managed forest stands, leaving 6673 plots that we classified into two climate regions (Mediterranean [i.e., 5244 plots] and temperate [i.e., 1429 plots]) based on the bioclimatic classification of Pesaresi et al. (2017). Although stand age and disturbance history are known to influence forest annual increment, the INFC does not include information on these properties. To assess potential influence of these factors on the 6673 plots included in our analyses, we compared stand‐level basal area at the beginning of the INFC census interval (as a proxy for forest age) with annual increment. These variables were only weakly correlated (Pearson's *r* = .22), suggesting no major bias in annual increment as a function of stand age in the plots used in our study. For each plot, we extracted climate data from 1980 to 2010 from the TerraClimate catalog with ~4 km^2^ spatial resolution (available at https://www.climatologylab.org/terraclimate.htm; Abatzoglou et al., 2018) using the ‘raster’ package (Hijmans, 2020) in R v. 4.1.3 (Figure S1; R Core Team, 2021). We focus on vapor pressure deficit (VPD, kPa) to represent a gradient of water limitation as it is an integrative measure of water stress that reflects the effects of both precipitation and temperature.

### 2.2 Functional trait data

To characterize the functional diversity of trees in INFC plots, we compiled publicly available data from the TRY (Kattge et al., 2020) and WOODIV (Monnet et al., 2021) databases. Note that a caveat of publicly available data is that we lack trait data for some rare species, however, the coverage of the trait data was high (see Appendix S1). We focused on the following traits: maximum tree height (m), seed mass (mg), wood density (g cm^−3^), specific leaf area (SLA; mm^2^ g^−1^), and xylem vulnerability to embolism measured as the xylem pressure inducing 50% loss of hydraulic conductivity due to embolism, that is, P_50_ (MPa) (Table S1). We chose these traits because they capture key variation in plant life‐history strategies (Costa‐Saura et al., 2019; Díaz et al., 2016), and are especially relevant to drought tolerance and species distributions along gradients of water limitation (Costa‐Saura et al., 2019; Maherali et al., 2004; Pinho et al., 2021; Trueba et al., 2017). Additionally, to characterize species multivariate phenotypes, we included the first axis of a Principal Component Analysis (PC1) based on the five individual traits using the R package “vegan” (Figure S2; Oksanen et al., 2020).

For each trait (including the multivariate trait axis, PC1), we calculated the plot‐level community‐weighted mean value (i.e., functional composition, CWM) and a measure of functional diversity (i.e., functional dispersion, FDis) using the R package “FD” (Laliberté & Legendre, 2010). We used species relative abundance to weigh both indices. FDis is generally independent of species richness (in our dataset, Pearson's *r* ranged from .04 to .15, depending on the trait; Figure S3). To compute FDis of species multivariate phenotypes, we included all five traits, allowing for one NA value per species (Table S2). Multivariate functional dispersion combines distance matrices of all five traits, if NA's were present within a certain trait, they were excluded before the construction of the trait‐specific distance matrices. Note that FDis computed without allowing NA values produces similar results but excludes more species (*r* = .78, *p* < .001).

### 2.3 Statistical analyses

Separately for each trait (including PC1), we used Structural Equation Modeling (SEM) to test mediation hypotheses for relationships among community functional properties (composition and dispersion) and annual increment. Our conceptual *a priori* model (Figure 1) is based on previous research designed to disentangle the relationships among ecosystem function, functional diversity, and community‐weighted mean traits (Chiang et al., 2016; Lohbeck et al., 2015). We applied structural equation modeling to the entire dataset, compiling a series of structural paths for each trait (i.e., seed mass, maximum height, SLA, wood density, P_50_, and PC1). Prior to analysis, all variables were log‐transformed (i.e., log(*x* + (1 − min(*x*)))), centered, and scaled to standard deviation to account for negative values, improve the symmetry of the distributions, and facilitate model fitting. A Wishart likelihood approach was used for the maximum likelihood (ML) estimation, and a full information maximum likelihood (FIML) method was used for missing data (Wothke, 1998). For each model, we first assessed model fit with root mean square error of approximation (RMSEA), the standardized root mean square residual (SRMR), the comparative fit index (CFI), and the Tucker–Lewis Index (TLI). We considered RMSEA <0.05, SRMR <0.08, CFI >0.95, and TLI >0.95 suggestive of good model fit (Browne & Cudeck, 1992; Hu & Bentler, 1999; Kline, 2010).

We applied Multigroup structural equation modeling (MG‐SEM) to determine if the proposed relationships among variables would vary across different bioclimatic regions, that is, temperate and Mediterranean. This multigroup analysis provides a direct test of measurement invariance between climatic groups, thus ensuring that the observed differences in structural relationships across conditions are unaffected by neither measurement errors nor measurement differences (see Appendix S1). SEMs were conducted using the “lavaan” package (Rosseel, 2012) implemented in the R environment v 4.1.2 (R Core Team, 2021).

## 3 RESULTS

Across Italy, the water limitation gradient (i.e., VPD) ranged from 0.14 to 0.90 kPa, while in the temperate climatic region it ranged from 0.14 to 0.78 kPa (mean 0.47 ± SD 0.12) and in the Mediterranean climate region it ranged from 0.36 to 0.90 kPa (mean 0.61 ± SD 0.09). Functional traits varied considerably across species (Table S1); PC1 explained 36.1% of the total trait variation and primarily represented a trade‐off between conservative and acquisitive resource strategies (i.e., negative correlation with SLA and height; Figure S2, Table 1). Forest annual increment varied substantially across Italy, ranging from 0.005 to 13.672 m^3^ ha^−1^ year^−1^.

### 3.1 Functional composition and dispersion along a climate gradient

**FIGURE 2:**
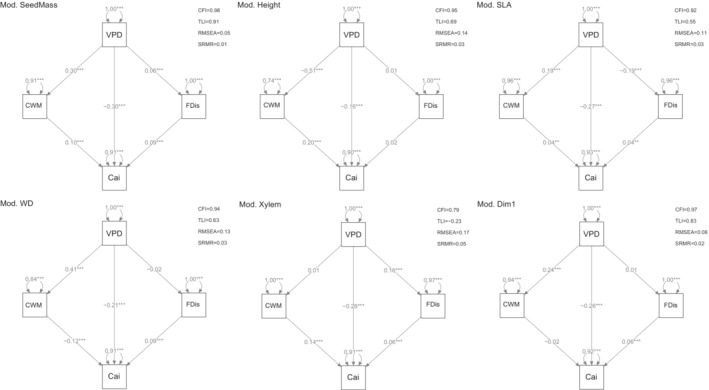
Results of structural equation modeling (SEM) on the pooled dataset for each functional trait; Seed mass (Mod. SeedMass), Height (Mod. Height), SLA (Mod. SLA), Wood density (Mod. WD), and Xylem vulnerability (Mod. Xylem) suit of traits, and the first PCA axis (Mod. Dim1). Arrowhead lines represent causal paths and bidirectional arrowhead indicates residual variance, with superimposed standardized partial regression coefficients: ***Significant (*p* < .05) paths. Squares represent manifest variables. At the top, model's fit indexes: CFI, comparative fit index; RMSEA, root mean square error of approximation index; SRMR, standardized root mean square residual; TLI, Tucker–Lewis index.

**FIGURE 3:**
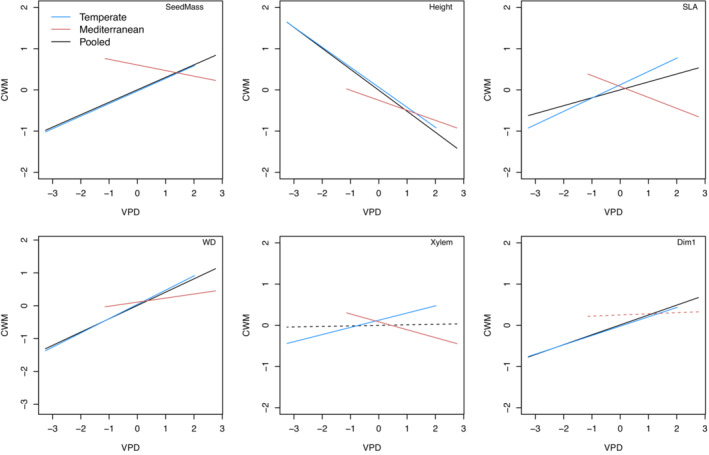
Results of Structural equation modeling (SEM) on the pooled dataset (black) and multigroup structural equation modeling (MG‐SEM) for the temperate (blue) and Mediterranean (red) bioclimatic domains. Lines represent the paths between community‐weighted mean (CWM) of seed mass (SeedMass), tree height (Height), specific leaf area (SLA), wood density (WD), xylem vulnerability (Xylem) function traits, and all traits (All) and a vapor pressure deficit (VPD); solid lines represent significant (*p* < .05) paths, dashed lines not significant ones.

**FIGURE 4:**
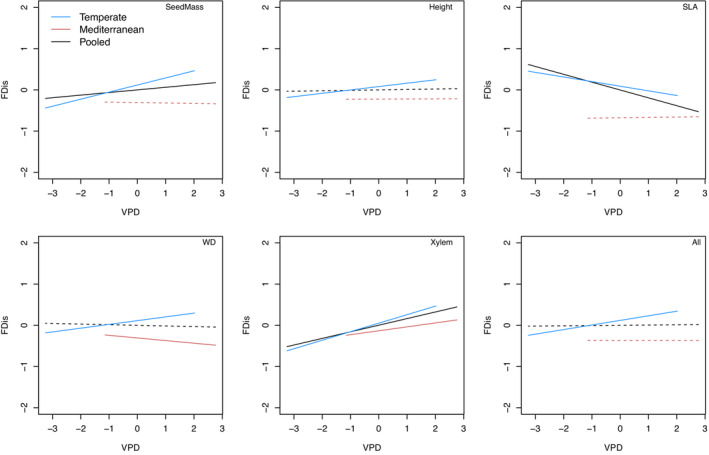
Results of Structural equation modeling (SEM) on the pooled dataset (black) and multigroup structural equation modeling (MG‐SEM) for the temperate (blue) and Mediterranean (red) bioclimatic domains. Lines represent the paths between functional dispersion (FDis) of seed mass (SeedMass), tree height (Height), specific leaf area (SLA), wood density (WD), xylem vulnerability (Xylem) function traits, and all traits (All) and a vapor pressure deficit (VPD) gradient; solid lines represent significant (*p* < .05) paths, dashed lines not significant ones.

The fit indices of specified SEM and MG‐SEM models, the former based on pooled data (Figure 2) and the latter on grouped data (Figures S4–S9), are well within the acceptable limits (Figure 2, Figures S4–S9). Patterns of functional composition and dispersion across the VPD gradient depended on spatial scale and varied between the climate regions (Figures 3 and 4, Tables S3–S14). At the national scale, CWM was positively associated with VPD for seed mass, SLA, wood density, and PC1 but negatively associated with VPD for maximum height. These trends were consistent when considering only the temperate forest plots and, in addition, CWM of P_50_ increased with VPD. Among plots in the Mediterranean climate region, VPD was negatively associated with CWM for seed mass, maximum height, SLA, and P_50_ and positively associated with CWM for wood density (Figure 3, Tables S3–S14). At the national scale, FDis was positively associated with VPD for seed mass and P_50_ but negatively associated with VPD for SLA. These trends were consistent for the temperate forest plots and, in addition, FDis of maximum height, wood density, and PC1 increased with VPD. Among plots in the Mediterranean climate region, VPD was negatively associated with FDis for wood density and positively associated with FDis for P_50_ (Figure 4, Tables S3–S14).

### 3.2 The link between functional composition, diversity, and annual increment

**FIGURE 5:**
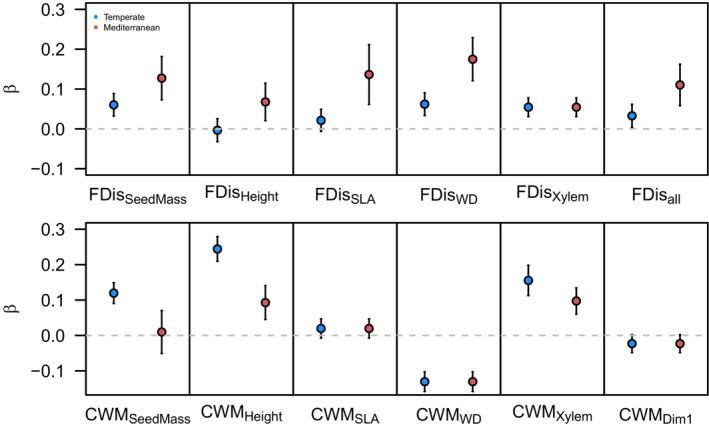
Results of multigroup structural equation modeling (MG‐SEM) for the temperate (blue) and Mediterranean (red) bioclimatic domains. Points and arrows represent the paths coefficient and 95% confidence interval between functional dispersion (FDis, upper panels) and community‐weighted mean (CWM, lower panels) of seed mass (SeedMass), tree height (Height), specific leaf area (SLA), wood density (WD), xylem vulnerability (Xylem) function traits, and all traits (All) and annual volume increment (Cai), respectively.

Paths linking annual increment to selected variables (i.e., VPD, CWM, and FDis) explained a total variance ranging from 7% to 10% based on pooled data and from 4% to 12% based on data grouped by climate region (Tables S3–S14). All parameter estimates and related fit indices are shown in Tables S3–S14. Our separate SEM models for each trait reveal that both CWM values and FDis were significantly related to annual increment for all traits, with the exceptions of maximum height, which was significantly related to annual increment only through CWM, and PC1, which was only significantly related to annual increment through FDis (Figure 2, Tables S3–S8). The multigroup structural equation models (MG‐SEM) for the Mediterranean and temperate climatic regions also support that CWM values and FDis significantly influenced annual increment, with some exceptions (Figure 5, Figures S4–S9, Tables S9–S14). However, the standardized path strengths between functional dispersion and annual increment were stronger among Mediterranean plots compared to temperate plots (Figure 5, Figures S4–S9, Tables S9–S14).

## 4 DISCUSSION

Improving our understanding of the relationship between biodiversity and ecosystem functioning is central to understanding the broad implications of community and ecosystem responses to environmental change (Gonzalez et al., 2020; Pires et al., 2018). Our results highlight that (i) functional composition of Italian forests is partly related to water limitation with a tendency for more conservative traits in the Mediterranean climate, (ii) the relationship between functional diversity and the VPD gradient varied between traits and bioclimatic regions, (iii) forest annual increment in Italian forests is positively associated with the diversity of resource strategies and the dominance of more acquisitive resource strategies, (iv) niche complementarity effects among plants species are increasingly important to forest annual increment under increased water limitation. Overall, our study suggests that environmental conditions (i.e., water limitation) can shape BEF relationships across large spatial scales by influencing patterns of dominance and the importance of niche complementarity effects.

### 4.1 Functional composition and diversity along a climate gradient

Although specific trait shifts along environmental gradients have commonly been observed (Costa‐Saura et al., 2016, 2017, 2019; Joswig et al., 2022; Pinho et al., 2021), we only found a consistent direction of functional composition shifts in the two climate regions for three traits (i.e., height, wood density, and PC1), highlighting the importance of spatial scale when assessing plant trait response to abiotic factors. In our study, opposite ends of the water limitation gradient likely face differing limiting factors. In the temperate climatic region, cold temperatures limit plant growth and development (Körner et al., 2016), and freezing stress can lead to mortality (Pittermann & Sperry, 2006). In the Mediterranean region, however, water limitation is a major limiting factor for plant growth and development (Gazol et al., 2018). The combined effect of cold stress and water limitation across the entire VPD gradient could help explain why we found a hump‐shaped response for certain traits (i.e., SLA, Seed mass, P_50_), with a shift toward coniferous species in cold stressed mountainous areas being the key factor behind more conservative trait values under low VPD in the temperate regions (Charrier et al., 2021; McCulloh et al., 2023).

Nevertheless, in the Mediterranean climatic region, we found that CWM traits tended to be more conservative in sites with higher levels of water limitation (higher VPD). As hypothesized, we found a trend of decreasing SLA, height, and P_50_, while stem density and PC1 increased with increasing water limitation (Costa‐Saura et al., 2016, 2017, 2019; Pinho et al., 2021). On the contrary, CWM of seed mass decreased with water limitation, which did not align with the hypothesis that larger seed size is beneficial for seedling survival under increased water limitation (Costa‐Saura et al., 2019; Metz et al., 2010; Volis & Bohrer, 2013). A possible explanation for this pattern could be the life‐history theory predictions for optimal seed size; for example, Larios and Venable (2018) showed that under water‐limited conditions when the cost of construction is considered, there is no overall fitness increase with seed size.

As with trends of CWM traits, variation of FDis along the VPD gradient also depended on the trait considered, the spatial scale of analysis, and the climate region of the plots. Nevertheless, we found a consistent direction of FDis shifts among the temperate plots, FDis was positively associated with VPD (i.e., higher functional diversity in more water‐limited sites) for seed mass, height, wood density, P_50,_ and PC1. At the same time, FDis of SLA was lower in sites with higher VPD. FDis of SLA is expected to be highest when coniferous and angiosperm species co‐occur due to large differences in leaf strategies (Maynard et al., 2022). This suggests that an increasing VPD is causing a shift toward the dominance of angiosperm species (Charrier et al., 2021; McCulloh et al., 2023). The FDis pattern of SLA along the VPD gradient confirms that the hump‐shaped trends for CWM traits, such as SLA, seed mass, and P_50_, result from a shift between coniferous and angiosperm species due to cold stress at lower VPD values. Additionally, an increase in FDis among the other five traits indicates a broad range of functional strategies that is able to co‐occur. The release of cold stress with increasing VPD in the temperate region leads to relatively benign environmental conditions, resulting in increased functional diversity.

### 4.2 Links between functional composition, diversity, and annual increment

Overall, our results support a positive relationship between functional diversity and annual increment (Cardinale et al., 2011; Chapin et al., 1997; Loreau et al., 2001). More specifically, we found evidence for niche complementarity effects through a positive association between FDis and site annual increment, suggesting that resource partitioning within tree communities positively influences annual increment in Italian forests. In contrast, maximum tree height was only associated with annual increment through mass ratio effects (CWM value), which aligns with previous research in that mass ratio effect of maximum height is strongly related with forest annual increment (Chiang et al., 2016; Conti & Díaz, 2013; Finegan et al., 2015). On the contrary, the direction of mass ratio effects varied among the traits; sites dominated by species with more acquisitive traits (e.g., increased height, SLA, and P_50_) showed higher annual increment, on average. Our results suggest that annual increment of Italian forests is positively influenced by a diversity of resource strategies that allow for resource partitioning, while at the same being positively associated with the dominance of tree species with more acquisitive resource strategies.

Drought is expected to increase in intensity and frequency in Italian forests (Spinoni et al., 2018), which could further alter the composition and annual increment of these forests. On one hand, we showed that those tree communities tended to be more conservative in sites with higher water limitation, while on the other hand, annual increment was positively influenced by the dominance of tree species with more acquisitive strategies, suggesting a decrease in forest annual increment in sites with higher water limitation due to a shift in functional composition. Under increased drought conditions, we expect a shift to forests dominated by species with relatively conservative traits, together with an associated decrease in forest annual increment, representing a challenge for future forest management.

### 4.3 BEF relationships across different bioclimatic regions

We found substantial differences in the mechanisms by which functional diversity influences annual increment between the climatic regions. We found a more predominant effect of niche complementarity (i.e., functional diversity) on annual increment in the Mediterranean climate region. In other words, competitively dominant species (i.e., mass ratio effects) appear to be less important to forest functioning under harsh conditions (i.e., increased water limitation), which is consistent with the prediction of the stress gradient hypothesis in that the frequency of competitive interactions will vary inversely across abiotic stress gradients (Paquette & Messier, 2011; Rita & Borghetti, 2019; Wang et al., 2019).

The fact that we found a stronger effect of niche complementarity (i.e., functional diversity) on annual increment in water‐limited plots could inform future forest management aiming to maintain annual increment under increasing drought (Spinoni et al., 2018). We showed that under increased water limitation, functional composition shifted to more conservative resource strategies, suggesting a decrease in forest annual increment. However, forest annual increment only showed a weak negative correlation with water limitation, shedding light on the importance of functional diversity for future forest management to maintain forest annual increment.

## AUTHOR CONTRIBUTIONS

**Roel Lammerant:** Conceptualization (equal); data curation (equal); formal analysis (equal); investigation (equal); methodology (equal); visualization (equal); writing – original draft (lead); writing – review and editing (lead). **Angelo Rita:** Conceptualization (equal); data curation (equal); formal analysis (equal); investigation (equal); methodology (equal); validation (equal); visualization (equal); writing – original draft (equal); writing – review and editing (equal). **Marco Borghetti:** Conceptualization (equal); formal analysis (equal); investigation (equal); methodology (equal); validation (equal); visualization (equal); writing – original draft (equal); writing – review and editing (equal). **Robert Muscarella:** Conceptualization (equal); formal analysis (equal); investigation (equal); methodology (equal); supervision (lead); validation (equal); visualization (equal); writing – original draft (equal); writing – review and editing (equal).

## CONFLICT OF INTEREST STATEMENT

The corresponding author confirms on behalf of all authors that there have been no involvements that might raise the question of bias in the work reported or in the conclusions, implications, or opinions stated.

## FUNDING INFORMATION

RM was supported by funding from the Swedish Research Council, Vetenskapsrådet (grant 2019‐03758).

## Supporting information


Appendix S1.

## Data Availability

The data used in our manuscript are already hosted in publicly available archives. Specifically, the Italian National Forest Inventory data is available on the INFC website (https://www.inventarioforestale.org/en). Functional trait data used in the study was downloaded from publicly available sources TRY (Kattge et al., 2020) and WOODIV (Monnet et al., 2021). Additionally, the r‐code that support the findings of this study are publicly available at https://github.com/bobmuscarella/INFC‐functional‐diversity.
