# Supplementary material for: Water‐limited environments affect the association between functional diversity and forest productivity
Source: Ecol Evol. 2023 Aug 8;13(8):e10406. doi: 10.1002/ece3.10406 (PMC10408253; doi:10.1002/ece3.10406)

**Supplementary materials**

**Title: Water-limited environments affect the association between functional diversity and forest productivity**

**Supplementary Methods**

***Model invariance across temperate and Mediterranean domains***

Testing for measurement invariance consisted of a series of statistical hypotheses that assume group factor parameters are equal between the groups. For multiple-group (MG) SEM models, the null hypothesis is that there are no group differences in any measurement parameters (Yuan and Chan, 2016). To this, a series of sequentially imposed measurement constraints, ranked as model with configural invariance (i.e. equal form), and model with strong invariance (i.e. equal intercepts) were performed as follows (see Vandenberg and Lance 2000, for description and review of main procedures). To test factorial invariance, we specified a succession of increasingly constrained measurement models for each trait. First, we evaluated a configural invariance model for each group with minimal constraints. In this model, with the exception of the reference items, all remaining factor loadings and intercepts were freely estimated. In the second level of invariance, we constrained the corresponding intercepts and path coefficients in each group to be invariant. The constrained and free models were formally compared via likelihood ratio chi-square difference test (Δ𝜒^2^); a particular level of invariance holds in the data when the Δ𝜒^2^ between two nested models is not significant (i.e., Δ𝜒^2^ in p< 0.05), meaning statistical model equivalence is supported. Then *i*) if the models were not significantly different, then the constrained model is equivalent to the free model i.e., the coefficients would not vary by group; *ii*) significant difference implies that some paths vary while others may not in the free and constrained models. In the latter case, we performed the process of introducing and releasing constraints in the model formula in order to identify which path varies between groups and re-fitting the model, namely finding partial Invariance (or individually constraining parameters). In detail, we repeatedly re-fitted a sequence of models ensuring each coefficient (under reasonable hypothesis) is equal between the two groups until no significant scaled Chi-squared difference between the two models was found, implying that the constrained model was valid. This approach was implemented using the library ‘piecewiseSEM’ with the function multigroup, which tests constraints and automatically selects the best output for our data. Improved or negligible changes in fit between configural and (partly)constrained models was required in order to conclude the level of invariance, specifically, CFI < 0.01, RMSEA < 0.015, and SRMR < 0.03.

**Supplementary Tables**

**Table S1**. Species coverage and range of traits included in functional diversity

| **Trait** | **Unit** | **Range** | **Species coverage** |
| --- | --- | --- | --- |
| Height | m | 1.50 to 50 | 86.86% |
| Seed mass | mg | 0.05 to 12478 | 89.78% |
| Wood density | g/cm^3^ | 0.28 to 0.84 | 87.59% |
| Specific leaf area (SLA) | mm^2^/mg | 0.51 to 29.88 | 82.48% |
| Xylem vulnerability (P50) | Mpa | -0.50 to -10.98 | 78,10% |

**Table S2**. Comparison of species, plot and measurement coverage for functional dispersion

| **Functional dispersion** | **Species coverage** | **Plot coverage** | **Individual measurements coverage** |
| --- | --- | --- | --- |
| 5 traits | 64.96% | 80.60% | 76.51% |
| 5 traits + 1 NA-value | 78.83% | 97.97% | 94.51% |

**Table S3**. Parameter estimates for the structural equation model (SeedMass) in Fig.2 of the main text. Est., estimated model parameters; Std.Err., standard errors for the free parameters in the model; z, z-statistic; p, p values corresponding to the z-statistic evaluated under a standard normal distribution; std, standardized model estimates. CWM, community weighted mean of seed mass; FDis, functional dispersion of seed mass; Cai, current annual increment, VPD, vapour pressure deficit.

|  | SeedMass | | | |
| --- | --- | --- | --- | --- |
|  | Est. | Std. Err. | z | p |
|  | Regression Slopes | | | |
| Cai |  |  |  |  |
| VPD | -0.30 | 0.01 | -23.42 | .000 |
| CWM | 0.10 | 0.01 | 7.41 | .000 |
| FDis | 0.09 | 0.01 | 7.00 | .000 |
| CWM |  |  |  |  |
| VPD | 0.30 | 0.01 | 25.43 | .000 |
| FDis |  |  |  |  |
| VPD | 0.06 | 0.01 | 5.16 | .000 |
|  | Intercepts | | | |
| Cai | 0.00 | 0.01 | 0.06 | .954 |
| FDis | 0.00 | 0.01 | 0.00 | .998 |
| CMW | 0.00 | 0.01 | 0.27 | .787 |
| VPD | -0.00 | 0.01 | -0.00 | 1.000 |
|  | Residual Variances | | | |
| Cai | 0.91 | 0.02 | 57.42 | .000 |
| CWM | 0.91 | 0.01 | 77.57 | .000 |
| FDis | 1.00 | 0.01 | 84.96 | .000 |
| VPD | 1.00 | 0.01 | 72.07 | .000 |
|  | Constructed | | | |
| be | 0.01 | 0.00 | 4.23 | .000 |
| ad | 0.03 | 0.00 | 7.12 | .000 |
| total | -0.27 | 0.01 | -21.32 | .000 |
|  | Fit Indices | | | |
| TLI | 0.91 |  |  |  |
| chisq | 19.99 |  |  |  |
| RMSEA | 0.05 |  |  |  |
| Scaled χ^2^ | 22.12(1) |  |  | 0 |
|  | R-squared | | | |
| Cai | 0.087 |  |  |  |
| CWM | 0.091 |  |  |  |
| FDis | 0.004 |  |  |  |

**Table S4**. Parameter estimates for the structural equation model (Height) in Fig.2 of the main text. Est., estimated model parameters; Std.Err., standard errors for the free parameters in the model; z, z-statistic; p, p values corresponding to the z-statistic evaluated under a standard normal distribution; std, standardized model estimates. CWM, community weighted mean of height; FDis, functional dispersion of height; Cai, current annual increment; VPD, vapour pressure deficit.

|  | Height | | | |
| --- | --- | --- | --- | --- |
|  | Est. | Std. Err. | z | P |
|  | Regression Slopes | | | |
| Cai |  |  |  |  |
| VPD | -0.16 | 0.01 | -11.21 | .000 |
| CMW | 0.20 | 0.01 | 13.92 | .000 |
| FDis | 0.02 | 0.01 | 1.52 | .128 |
| CMW |  |  |  |  |
| VPD | -0.51 | 0.01 | -45.10 | .000 |
| FDis |  |  |  |  |
| VPD | 0.01 | 0.01 | 0.80 | .421 |
|  | Intercepts | | | |
| Cai | 0.00 | 0.01 | 0.07 | .943 |
| CMW | -0.01 | 0.01 | -0.79 | .429 |
| FDis | -0.00 | 0.01 | -0.00 | .999 |
| VPD | -0.00 | 0.01 | -0.00 | 1.000 |
|  | Residual Variances | | | |
| Cai | 0.90 | 0.02 | 58.28 | .000 |
| CMW | 0.74 | 0.01 | 50.67 | .000 |
| FDis | 1.00 | 0.02 | 58.27 | .000 |
| VPD | 1.00 | 0.01 | 72.07 | .000 |
|  | Constructed | | | |
| be | 0.00 | 0.00 | 0.71 | .478 |
| ad | -0.10 | 0.01 | -13.39 | .000 |
| total | -0.27 | 0.01 | -21.25 | .000 |
|  | Fit Indices | | | |
| TLI | 0.69 |  |  |  |
| chisq | 139.90 |  |  |  |
| RMSEA | 0.14 |  |  |  |
| Scaled χ^2^ | 130.93(1) |  |  | 0 |
|  | R-squared | | | |
| Cai | 0.100 |  |  |  |
| CMW | 0.259 |  |  |  |
| FDis | 0.001 |  |  |  |

**Table S5**. Parameter estimates for the structural equation model (SLA) in Fig. 2 of the main text. Est., estimated model parameters; Std.Err., standard errors for the free parameters in the model; z, z-statistic; p, p values corresponding to the z-statistic evaluated under a standard normal distribution; std, standardized model estimates. CWM, community weighted mean of specific leaf area; FDis, functional dispersion of specific leaf area; Cai, current annual increment; VPD, vapour pressure deficit.

|  | SLA | | | | |
| --- | --- | --- | --- | --- | --- |
|  | Est. | | Std. Err. | z | p |
|  | Regression Slopes | | | | |
| Cai |  | |  |  |  |
| VPD | -0.27 | | 0.01 | -20.56 | 0 |
| CMW | 0.04 | | 0.01 | 2.95 | 0.003 |
| FDis | 0.04 | | 0.01 | 3.27 | 0.001 |
| CMW |  | |  |  |  |
| VPD | 0.19 | | 0.01 | 13.29 | 0 |
| FDis |  | |  |  |  |
| VPD | -0.19 | | 0.01 | -14.46 | 0 |
|  | Intercepts | | | | |
| Cai | 0 | | 0.01 | 0.04 | 0.97 |
| CMW | 0 | | 0.01 | 0.15 | 0.884 |
| FDis | 0 | | 0.01 | -0.22 | 0.824 |
| VPD | 0 | | 0.01 | 0 | 1 |
|  | Residual Variances | | | | |
| Cai | 0.93 | | 0.02 | 58.07 | 0 |
| CMW | 0.96 | | 0.02 | 39.82 | 0 |
| FDis | 0.96 | | 0.02 | 59.53 | 0 |
| VPD | 1 | | 0.01 | 72.07 | 0 |
|  | Constructed | | | | |
| be | -0.01 | | 0 | -3.19 | 0.001 |
| ad | 0.01 | | 0 | 2.89 | 0.004 |
| total | -0.27 | | 0.01 | -21.28 | 0 |
|  | Fit Indices | | | | |
| TLI | 0.55 | |  |  |  |
| chisq | 76.98 | |  |  |  |
| RMSEA | 0.11 | |  |  |  |
| Scaled χ^2^ | 56.89(1) | |  |  | 0 |
|  | | R-squared | | | |
| Cai | 0.074 | |  |  |  |
| CMW | 0.037 | |  |  |  |
| FDis | 0.036 | |  |  |  |

**Table S6**. Parameter estimates for the structural equation model (WD) in Fig. 2 of the main text. Est., estimated model parameters; Std.Err., standard errors for the free parameters in the model; z, z-statistic; p, p values corresponding to the z-statistic evaluated under a standard normal distribution; std, standardized model estimates. CWM, community weighted mean of wood density; FDis, functional dispersion of wood density; Cai, current annual increment; VPD, vapour pressure deficit.

|  | WD | | | | | |
| --- | --- | --- | --- | --- | --- | --- |
|  | Est. | | Std. Err. | z | P | |
|  | Regression Slopes | | | | | |
| Cai |  | |  |  |  | |
| VPD | -0.21 | | 0.01 | -15.27 | 0 | |
| CMW | -0.12 | | 0.01 | -8.85 | 0 | |
| FDis | 0.09 | | 0.01 | 7.36 | 0 | |
| CMW |  | |  |  |  | |
| VPD | 0.41 | | 0.01 | 36.18 | 0 | |
| FDis |  | |  |  |  | |
| VPD | -0.02 | | 0.01 | -1.28 | 0.2 | |
|  | Intercepts | | | | | |
| Cai | 0 | | 0.01 | 0.09 | 0.928 | |
| CMW | 0.01 | | 0.01 | 0.67 | 0.5 | |
| FDis | 0 | | 0.01 | -0.1 | 0.924 | |
| VPD | 0 | | 0.01 | 0 | 1 | |
|  | Residual Variances | | | | | |
| Cai | 0.91 | | 0.02 | 58.29 | 0 | |
| CMW | 0.84 | | 0.01 | 68.57 | 0 | |
| FDis | 1 | | 0.01 | 73.93 | 0 | |
| VPD | 1 | | 0.01 | 72.07 | 0 | |
|  | Constructed | | | | | |
| be | 0 | | 0 | -1.26 | 0.209 | |
| ad | -0.05 | | 0.01 | -8.47 | 0 | |
| total | -0.27 | | 0.01 | -21.31 | 0 | |
|  | Fit Indices | | | | | |
| TLI | 0.63 | |  |  |  | |
| chisq | 114 | |  |  |  | |
| RMSEA | 0.13 | |  |  |  | |
| Scaled χ^2^ | 129.79(1) | |  |  | 0 | |
|  | | R-squared | | | |  |
| Cai | 0.093 | |  |  |  | |
| CMW | 0.164 | |  |  |  | |
| FDis | 0.001 | |  |  |  | |

**Table S7**. Parameter estimates for the structural equation model (Xylem) in Fig. 2 of the main text. Est., estimated model parameters; Std.Err., standard errors for the free parameters in the model; z, z-statistic; p, p values corresponding to the z-statistic evaluated under a standard normal distribution; std, standardized model estimates. CWM, community weighted mean of xylem vulnerability; FDis, functional dispersion of xylem vulnerability; Cai, current annual increment; VPD, vapour pressure deficit.

|  | Xylem | | | | | |
| --- | --- | --- | --- | --- | --- | --- |
|  | Est. | | Std. Err. | z | p | |
|  | Regression Slopes | | | | | |
| Cai |  | |  |  |  | |
| VPD | -0.28 | | 0.01 | -22.17 | 0 | |
| CMW | 0.14 | | 0.02 | 9.12 | 0 | |
| FDis | 0.06 | | 0.01 | 4.99 | 0 | |
| CMW |  | |  |  |  | |
| VPD | 0.01 | | 0.01 | 0.92 | 0.357 | |
| FDis |  | |  |  |  | |
| VPD | 0.16 | | 0.01 | 12.79 | 0 | |
|  | Intercepts | | | | | |
| Cai | 0 | | 0.01 | 0.11 | 0.914 | |
| CMW | 0 | | 0.01 | -0.12 | 0.906 | |
| FDis | 0 | | 0.01 | 0.19 | 0.846 | |
| VPD | 0 | | 0.01 | 0 | 1 | |
|  | Residual Variances | | | | | |
| Cai | 0.91 | | 0.02 | 58.09 | 0 | |
| CMW | 1 | | 0.11 | 9.02 | 0 | |
| FDis | 0.97 | | 0.03 | 32.55 | 0 | |
| VPD | 1 | | 0.01 | 72.07 | 0 | |
|  | Constructed | | | | | |
| be | 0.01 | | 0 | 4.74 | 0 | |
| ad | 0 | | 0 | 0.88 | 0.381 | |
| total | -0.27 | | 0.01 | -21.24 | 0 | |
|  | Fit Indices | | | | | |
| TLI | -0.23 | |  |  |  | |
| chisq | 192 | |  |  |  | |
| RMSEA | 0.17 | |  |  |  | |
| Scaled χ^2^ | 34.30(1) | |  |  | 0 | |
|  | | R-squared | | | |  |
| Cai | 0.093 | |  |  |  | |
| CMW | 0 | |  |  |  | |
| FDis | 0.026 | |  |  |  | |

**Table S8**. Parameter estimates for the structural equation model (Dim1) in Fig. 2 of the main text. Est., estimated model parameters; Std.Err., standard errors for the free parameters in the model; z, z-statistic; p, p values corresponding to the z-statistic evaluated under a standard normal distribution; std, standardized model estimates. CWM, community weighted mean of PCA1 axis; FDis, functional dispersion of PCA1 axis; Cai, current annual increment; VPD, vapour pressure deficit.

|  | Dim1 | | | | | |
| --- | --- | --- | --- | --- | --- | --- |
|  | Est. | | Std. Err. | z | P | |
|  | Regression Slopes | | | | | |
| Cai |  | |  |  |  | |
| VPD | -0.26 | | 0.01 | -20.29 | 0 | |
| CMW | -0.02 | | 0.01 | -1.86 | 0.063 | |
| FDis | 0.06 | | 0.01 | 4.65 | 0 | |
| CMW |  | |  |  |  | |
| VPD | 0.24 | | 0.01 | 21.06 | 0 | |
| FDis |  | |  |  |  | |
| VPD | 0.01 | | 0.01 | 0.48 | 0.628 | |
|  | Intercepts | | | | | |
| Cai | 0 | | 0.01 | 0.05 | 0.957 | |
| CMW | 0.01 | | 0.01 | 0.78 | 0.436 | |
| FDis | 0 | | 0.01 | -0.03 | 0.973 | |
| VPD | 0 | | 0.01 | 0 | 1 | |
|  | Residual Variances | | | | | |
| Cai | 0.93 | | 0.02 | 58.06 | 0 | |
| CMW | 0.94 | | 0.02 | 53.37 | 0 | |
| FDis | 1 | | 0.01 | 84.43 | 0 | |
| VPD | 1 | | 0.01 | 72.07 | 0 | |
|  | Constructed | | | | | |
| be | 0 | | 0 | 0.48 | 0.629 | |
| ad | -0.01 | | 0 | -1.84 | 0.066 | |
| total | -0.27 | | 0.01 | -21.29 | 0 | |
|  | Fit Indices | | | | | |
| TLI | 0.83 | |  |  |  | |
| chisq | 25.93 | |  |  |  | |
| RMSEA | 0.06 | |  |  |  | |
| Scaled χ^2^ | 25.49(1) | |  |  | 0 | |
|  | | R-squared | | | |  |
| Cai | 0.075 | |  |  |  | |
| CMW | 0.058 | |  |  |  | |
| FDis | 0 | |  |  |  | |

**Table S9**. Parameter estimates of the partly constrained multigroup structural equation model (SeedMass construct) showed in Fig. S4. The paths VPD˜Cai and CWM˜Cai were constrained to be invariant between groups. lhs, left-hand-side; op, operators: means is regressed onto; means is corelated with; ˜1 means intercept; rhs, right-hand-side; est, estimated model parameters; se, standard errors for the free parameters in the model; z, value of the estimate divided by its standard error (so-called z-statistic); ci.lower and ci.upper, lower 2.5% and upper 97.5% confidence intervals of z-statistic; p, p values corresponding to the z-statistic evaluated under a standard normal distribution; std, standardized model estimates.

| **lhs** | **op** | **rhs** | **block** | **group** | **label** | **est** | **se** | **z** | **pvalue** | **ci.lower** | **ci.upper** | **std.lv** | **std.all** |
| --- | --- | --- | --- | --- | --- | --- | --- | --- | --- | --- | --- | --- | --- |
| Cai | ~ | VPD | 1 | 1 | c0 | -0.26 | 0.02 | -16.72 | 0 | -0.29 | -0.23 | -0.26 | -0.25 |
| Cai | ~ | CWM | 1 | 1 | d0 | 0.12 | 0.01 | 8 | 0 | 0.09 | 0.15 | 0.12 | 0.12 |
| Cai | ~ | FDis | 1 | 1 | e0 | 0.06 | 0.01 | 4.18 | 0 | 0.03 | 0.09 | 0.06 | 0.06 |
| CWM | ~ | VPD | 1 | 1 | a0 | 0.32 | 0.01 | 23.57 | 0 | 0.29 | 0.34 | 0.32 | 0.31 |
| FDis | ~ | VPD | 1 | 1 | b0 | 0.18 | 0.01 | 12.97 | 0 | 0.15 | 0.21 | 0.18 | 0.17 |
| Cai | ~1 |  | 1 | 1 |  | 0.04 | 0.01 | 2.75 | 0.01 | 0.01 | 0.07 | 0.04 | 0.04 |
| FDis | ~1 |  | 1 | 1 |  | 0.12 | 0.01 | 8.46 | 0 | 0.09 | 0.14 | 0.12 | 0.12 |
| CWM | ~1 |  | 1 | 1 |  | -0.03 | 0.01 | -2.02 | 0.04 | -0.05 | 0 | -0.03 | -0.03 |
| VPD | ~1 |  | 1 | 1 |  | -0.23 | 0.01 | -17.36 | 0 | -0.26 | -0.2 | -0.23 | -0.24 |
| Cai | ~~ | Cai | 1 | 1 |  | 0.92 | 0.02 | 52.07 | 0 | 0.88 | 0.95 | 0.92 | 0.94 |
| CWM | ~~ | CWM | 1 | 1 |  | 0.9 | 0.01 | 70.2 | 0 | 0.87 | 0.92 | 0.9 | 0.91 |
| FDis | ~~ | FDis | 1 | 1 |  | 0.96 | 0.01 | 73.41 | 0 | 0.93 | 0.98 | 0.96 | 0.97 |
| VPD | ~~ | VPD | 1 | 1 |  | 0.91 | 0.01 | 64.61 | 0 | 0.89 | 0.94 | 0.91 | 1 |
| Cai | ~ | VPD | 2 | 2 | c1 | -0.43 | 0.05 | -9.52 | 0 | -0.52 | -0.34 | -0.43 | -0.28 |
| Cai | ~ | CWM | 2 | 2 | d1 | 0.01 | 0.03 | 0.31 | 0.75 | -0.05 | 0.07 | 0.01 | 0.01 |
| Cai | ~ | FDis | 2 | 2 | e1 | 0.13 | 0.03 | 4.59 | 0 | 0.07 | 0.18 | 0.13 | 0.13 |
| CWM | ~ | VPD | 2 | 2 | a1 | -0.2 | 0.04 | -4.79 | 0 | -0.28 | -0.12 | -0.2 | -0.14 |
| FDis | ~ | VPD | 2 | 2 | b1 | -0.02 | 0.04 | -0.38 | 0.7 | -0.1 | 0.07 | -0.02 | -0.01 |
| Cai | ~1 |  | 2 | 2 |  | 0.06 | 0.05 | 1.19 | 0.24 | -0.04 | 0.17 | 0.06 | 0.07 |
| FDis | ~1 |  | 2 | 2 |  | -0.3 | 0.05 | -6.51 | 0 | -0.39 | -0.21 | -0.3 | -0.31 |
| CWM | ~1 |  | 2 | 2 |  | 0.56 | 0.04 | 12.87 | 0 | 0.47 | 0.64 | 0.56 | 0.61 |
| VPD | ~1 |  | 2 | 2 |  | 0.85 | 0.02 | 50.63 | 0 | 0.82 | 0.89 | 0.85 | 1.35 |
| Cai | ~~ | Cai | 2 | 2 |  | 0.86 | 0.04 | 24.61 | 0 | 0.79 | 0.93 | 0.86 | 0.91 |
| CWM | ~~ | CWM | 2 | 2 |  | 0.84 | 0.03 | 27.97 | 0 | 0.78 | 0.89 | 0.84 | 0.98 |
| FDis | ~~ | FDis | 2 | 2 |  | 0.94 | 0.03 | 31.88 | 0 | 0.88 | 0.99 | 0.94 | 1 |
| VPD | ~~ | VPD | 2 | 2 |  | 0.4 | 0.02 | 26.17 | 0 | 0.37 | 0.43 | 0.4 | 1 |
| b0e0 | := | b0*e0 | 0 | 0 | b0e0 | 0.01 | 0 | 4.01 | 0 | 0.01 | 0.02 | 0.01 | 0.01 |
| a0d0 | := | a0*d0 | 0 | 0 | a0d0 | 0.04 | 0.01 | 7.55 | 0 | 0.03 | 0.05 | 0.04 | 0.04 |
| total0 | := | c0+(b0*e0)+(a0*d0) | 0 | 0 | total0 | -0.21 | 0.01 | -14.36 | 0 | -0.24 | -0.18 | -0.21 | -0.2 |
| b1e1 | := | b1*e1 | 0 | 0 | b1e1 | 0 | 0.01 | -0.38 | 0.7 | -0.01 | 0.01 | 0 | 0 |
| a1d0 | := | a1*d0 | 0 | 0 | a1d1 | 0 | 0.01 | -0.31 | 0.76 | -0.01 | 0.01 | 0 | 0 |
| total1 | := | c0+(b1*e1)+(a1*d0) | 0 | 0 | total1 | -0.43 | 0.04 | -9.74 | 0 | -0.52 | -0.35 | -0.43 | -0.28 |

**Table S10**. Parameter estimates from the multigroup structural equation model (Height construct) showed in Fig. S5. Any paths were constrained to be invariant between groups as constrained model showed performance compared to unconstrained one. lhs, left-hand-side; op, operators: means is regressed onto; means is corelated with; ˜1 means intercept; rhs, right-hand-side; est, estimated model parameters; se, standard errors for the free parameters in the model; z, value of the estimate divided by its standard error (so-called z-statistic); ci.lower and ci.upper, lower 2.5% and upper 97.5% confidence intervals of z-statistic; p, p values corresponding to the z-statistic evaluated under a standard normal distribution; std, standardized model estimates.

| **lhs** | **op** | **rhs** | **block** | **group** | **label** | **est** | **se** | **z** | **pvalue** | **ci.lower** | **ci.upper** | **std.lv** | **std.all** |
| --- | --- | --- | --- | --- | --- | --- | --- | --- | --- | --- | --- | --- | --- |
| Cai | ~ | VPD | 1 | 1 | c0 | -0.1 | 0.02 | -5.85 | 0 | -0.13 | -0.07 | -0.1 | -0.1 |
| Cai | ~ | CWM | 1 | 1 | d0 | 0.24 | 0.02 | 13.7 | 0 | 0.21 | 0.28 | 0.24 | 0.22 |
| Cai | ~ | FDis | 1 | 1 | e0 | 0 | 0.01 | -0.24 | 0.81 | -0.03 | 0.03 | 0 | 0 |
| CWM | ~ | VPD | 1 | 1 | a0 | -0.46 | 0.01 | -38.27 | 0 | -0.48 | -0.43 | -0.46 | -0.49 |
| FDis | ~ | VPD | 1 | 1 | b0 | 0.08 | 0.01 | 5.62 | 0 | 0.05 | 0.11 | 0.08 | 0.08 |
| Cai | ~1 |  | 1 | 1 |  | 0.03 | 0.01 | 1.96 | 0.05 | 0 | 0.06 | 0.03 | 0.03 |
| FDis | ~1 |  | 1 | 1 |  | 0.08 | 0.01 | 5.7 | 0 | 0.05 | 0.11 | 0.08 | 0.08 |
| CWM | ~1 |  | 1 | 1 |  | 0.06 | 0.01 | 5.23 | 0 | 0.04 | 0.08 | 0.06 | 0.06 |
| VPD | ~1 |  | 1 | 1 |  | -0.23 | 0.01 | -17.36 | 0 | -0.26 | -0.2 | -0.23 | -0.24 |
| Cai | ~~ | Cai | 1 | 1 |  | 0.9 | 0.02 | 52.21 | 0 | 0.86 | 0.93 | 0.9 | 0.92 |
| CWM | ~~ | CWM | 1 | 1 |  | 0.61 | 0.01 | 48.61 | 0 | 0.59 | 0.64 | 0.61 | 0.76 |
| FDis | ~~ | FDis | 1 | 1 |  | 0.93 | 0.02 | 54.82 | 0 | 0.9 | 0.96 | 0.93 | 0.99 |
| VPD | ~~ | VPD | 1 | 1 |  | 0.91 | 0.01 | 64.61 | 0 | 0.89 | 0.94 | 0.91 | 1 |
| Cai | ~ | VPD | 2 | 2 | c1 | -0.39 | 0.04 | -8.71 | 0 | -0.48 | -0.3 | -0.39 | -0.25 |
| Cai | ~ | CWM | 2 | 2 | d1 | 0.09 | 0.02 | 3.81 | 0 | 0.04 | 0.14 | 0.09 | 0.11 |
| Cai | ~ | FDis | 2 | 2 | e1 | 0.07 | 0.02 | 2.83 | 0 | 0.02 | 0.11 | 0.07 | 0.08 |
| CWM | ~ | VPD | 2 | 2 | a1 | -0.43 | 0.04 | -10.02 | 0 | -0.52 | -0.35 | -0.43 | -0.24 |
| FDis | ~ | VPD | 2 | 2 | b1 | 0.01 | 0.05 | 0.11 | 0.91 | -0.09 | 0.1 | 0.01 | 0 |
| Cai | ~1 |  | 2 | 2 |  | 0.07 | 0.05 | 1.38 | 0.17 | -0.03 | 0.17 | 0.07 | 0.07 |
| FDis | ~1 |  | 2 | 2 |  | -0.24 | 0.05 | -4.98 | 0 | -0.34 | -0.15 | -0.24 | -0.22 |
| CWM | ~1 |  | 2 | 2 |  | -0.28 | 0.04 | -6.38 | 0 | -0.37 | -0.2 | -0.28 | -0.25 |
| VPD | ~1 |  | 2 | 2 |  | 0.85 | 0.02 | 50.63 | 0 | 0.82 | 0.89 | 0.85 | 1.35 |
| Cai | ~~ | Cai | 2 | 2 |  | 0.86 | 0.03 | 25.19 | 0 | 0.8 | 0.93 | 0.86 | 0.91 |
| CWM | ~~ | CWM | 2 | 2 |  | 1.2 | 0.05 | 26.41 | 0 | 1.11 | 1.29 | 1.2 | 0.94 |
| FDis | ~~ | FDis | 2 | 2 |  | 1.18 | 0.06 | 20.57 | 0 | 1.07 | 1.29 | 1.18 | 1 |
| VPD | ~~ | VPD | 2 | 2 |  | 0.4 | 0.02 | 26.17 | 0 | 0.37 | 0.43 | 0.4 | 1 |
| b0e0 | := | b0*e0 | 0 | 0 | b0e0 | 0 | 0 | -0.24 | 0.81 | 0 | 0 | 0 | 0 |
| a0d0 | := | a0*d0 | 0 | 0 | a0d0 | -0.11 | 0.01 | -12.91 | 0 | -0.13 | -0.09 | -0.11 | -0.11 |
| total0 | := | c0+(b0*e0)+(a0*d0) | 0 | 0 | total0 | -0.21 | 0.01 | -14.29 | 0 | -0.24 | -0.18 | -0.21 | -0.2 |
| b1e1 | := | b1*e1 | 0 | 0 | b1e1 | 0 | 0 | 0.11 | 0.91 | -0.01 | 0.01 | 0 | 0 |
| a1d1 | := | a1*d1 | 0 | 0 | a1d1 | -0.04 | 0.01 | -3.57 | 0 | -0.06 | -0.02 | -0.04 | -0.03 |
| total1 | := | c1+(b1*e1)+(a1*d1) | 0 | 0 | total1 | -0.43 | 0.04 | -9.66 | 0 | -0.52 | -0.34 | -0.43 | -0.28 |

**Table S11**. Parameter estimates of the partly constrained multigroup structural equation model (SLA construct) showed in Fig. S6. The path CWM˜Cai was constrained to be invariant between groups. lhs, left-hand-side; op, operators: means is regressed onto; means is corelated with; ˜1 means intercept; rhs, right-hand-side; est, estimated model parameters; se, standard errors for the free parameters in the model; z, value of the estimate divided by its standard error (so-called z-statistic); ci.lower and ci.upper, lower 2.5% and upper 97.5% confidence intervals of z-statistic; p, p values corresponding to the z-statistic evaluated under a standard normal distribution; std, standardized model estimates.

| **lhs** | **op** | **rhs** | **block** | **group** | **label** | **est** | **se** | **z** | **pvalue** | **ci.lower** | **ci.upper** | **std.lv** | **std.all** |
| --- | --- | --- | --- | --- | --- | --- | --- | --- | --- | --- | --- | --- | --- |
| Cai | ~ | VPD | 1 | 1 | c0 | -0.22 | 0.02 | -13.93 | 0 | -0.25 | -0.19 | -0.22 | -0.21 |
| Cai | ~ | CWM | 1 | 1 | d0 | 0.02 | 0.01 | 1.43 | 0.15 | -0.01 | 0.05 | 0.02 | 0.02 |
| Cai | ~ | FDis | 1 | 1 | e0 | 0.02 | 0.01 | 1.5 | 0.13 | -0.01 | 0.05 | 0.02 | 0.02 |
| CWM | ~ | VPD | 1 | 1 | a0 | 0.35 | 0.02 | 22.21 | 0 | 0.32 | 0.39 | 0.35 | 0.32 |
| FDis | ~ | VPD | 1 | 1 | b0 | -0.12 | 0.02 | -7.85 | 0 | -0.15 | -0.09 | -0.12 | -0.11 |
| Cai | ~1 |  | 1 | 1 |  | 0.04 | 0.01 | 2.66 | 0.01 | 0.01 | 0.07 | 0.04 | 0.04 |
| FDis | ~1 |  | 1 | 1 |  | 0.09 | 0.01 | 6.78 | 0 | 0.06 | 0.12 | 0.09 | 0.09 |
| CWM | ~1 |  | 1 | 1 |  | 0.13 | 0.01 | 10.59 | 0 | 0.11 | 0.15 | 0.13 | 0.12 |
| VPD | ~1 |  | 1 | 1 |  | -0.23 | 0.01 | -17.36 | 0 | -0.26 | -0.2 | -0.23 | -0.24 |
| Cai | ~~ | Cai | 1 | 1 |  | 0.93 | 0.02 | 52.69 | 0 | 0.9 | 0.97 | 0.93 | 0.96 |
| CWM | ~~ | CWM | 1 | 1 |  | 0.98 | 0.03 | 34.9 | 0 | 0.93 | 1.04 | 0.98 | 0.9 |
| FDis | ~~ | FDis | 1 | 1 |  | 1.04 | 0.02 | 55.92 | 0 | 1 | 1.08 | 1.04 | 0.99 |
| VPD | ~~ | VPD | 1 | 1 |  | 0.91 | 0.01 | 64.61 | 0 | 0.89 | 0.94 | 0.91 | 1 |
| Cai | ~ | VPD | 2 | 2 | c1 | -0.43 | 0.04 | -9.54 | 0 | -0.51 | -0.34 | -0.43 | -0.28 |
| Cai | ~ | CWM | 2 | 2 | d0 | 0.02 | 0.01 | 1.43 | 0.15 | -0.01 | 0.05 | 0.02 | 0.02 |
| Cai | ~ | FDis | 2 | 2 | e1 | 0.14 | 0.04 | 3.56 | 0 | 0.06 | 0.21 | 0.14 | 0.1 |
| CWM | ~ | VPD | 2 | 2 | a1 | -0.31 | 0.03 | -10.61 | 0 | -0.37 | -0.26 | -0.31 | -0.27 |
| FDis | ~ | VPD | 2 | 2 | b1 | 0.01 | 0.03 | 0.29 | 0.77 | -0.06 | 0.08 | 0.01 | 0.01 |
| Cai | ~1 |  | 2 | 2 |  | 0.09 | 0.05 | 1.72 | 0.08 | -0.01 | 0.2 | 0.09 | 0.09 |
| FDis | ~1 |  | 2 | 2 |  | -0.48 | 0.04 | -13.43 | 0 | -0.55 | -0.41 | -0.48 | -0.68 |
| CWM | ~1 |  | 2 | 2 |  | 0.06 | 0.03 | 2.13 | 0.03 | 0 | 0.12 | 0.06 | 0.08 |
| VPD | ~1 |  | 2 | 2 |  | 0.85 | 0.02 | 50.63 | 0 | 0.82 | 0.89 | 0.85 | 1.35 |
| Cai | ~~ | Cai | 2 | 2 |  | 0.87 | 0.04 | 24.75 | 0 | 0.8 | 0.94 | 0.87 | 0.91 |
| CWM | ~~ | CWM | 2 | 2 |  | 0.52 | 0.02 | 26.65 | 0 | 0.48 | 0.56 | 0.52 | 0.93 |
| FDis | ~~ | FDis | 2 | 2 |  | 0.51 | 0.03 | 17.69 | 0 | 0.46 | 0.57 | 0.51 | 1 |
| VPD | ~~ | VPD | 2 | 2 |  | 0.4 | 0.02 | 26.17 | 0 | 0.37 | 0.43 | 0.4 | 1 |
| b0e0 | := | b0*e0 | 0 | 0 | b0e0 | 0 | 0 | -1.48 | 0.14 | -0.01 | 0 | 0 | 0 |
| a0d0 | := | a0*d0 | 0 | 0 | a0d0 | 0.01 | 0 | 1.43 | 0.15 | 0 | 0.02 | 0.01 | 0.01 |
| total0 | := | c0+(b0*e0)+(a0*d0) | 0 | 0 | total0 | -0.21 | 0.01 | -14.34 | 0 | -0.24 | -0.18 | -0.21 | -0.2 |
| b1e1 | := | b1*e1 | 0 | 0 | b1e1 | 0 | 0 | 0.29 | 0.77 | -0.01 | 0.01 | 0 | 0 |
| a1d0 | := | a1*d0 | 0 | 0 | a1d0 | -0.01 | 0 | -1.41 | 0.16 | -0.01 | 0 | -0.01 | -0.01 |
| total1 | := | c1+(b1*e1)+(a1*d0) | 0 | 0 | total1 | -0.43 | 0.04 | -9.67 | 0 | -0.52 | -0.34 | -0.43 | -0.28 |

**Table S12**. Parameter estimates from the multigroup structural equation model (WD construct) showed in Fig. S7. Any paths were constrained to be invariant between groups as constrained model showed performance compared to unconstrained one. lhs, left-hand-side; op, operators: means is regressed onto; means is corelated with; ˜1 means intercept; rhs, right-hand-side; est, estimated model parameters; se, standard errors for the free parameters in the model; z, value of the estimate divided by its standard error (so-called z-statistic); ci.lower and ci.upper, lower 2.5% and upper 97.5% confidence intervals of z-statistic; p, p values corresponding to the z-statistic evaluated under a standard normal distribution; std, standardized model estimates.

| **lhs** | **op** | **rhs** | **block** | **group** | **label** | **est** | **se** | **z** | **pvalue** | **ci.lower** | **ci.upper** | **std.lv** | **std.all** |
| --- | --- | --- | --- | --- | --- | --- | --- | --- | --- | --- | --- | --- | --- |
| Cai | ~ | VPD | 1 | 1 | c0 | -0.16 | 0.02 | -9.6 | 0 | -0.19 | -0.13 | -0.16 | -0.15 |
| Cai | ~ | CWM | 1 | 1 | d0 | -0.13 | 0.01 | -9.2 | 0 | -0.16 | -0.1 | -0.13 | -0.13 |
| Cai | ~ | FDis | 1 | 1 | e0 | 0.06 | 0.01 | 4.25 | 0 | 0.03 | 0.09 | 0.06 | 0.06 |
| CWM | ~ | VPD | 1 | 1 | a0 | 0.45 | 0.01 | 36.36 | 0 | 0.43 | 0.48 | 0.45 | 0.43 |
| FDis | ~ | VPD | 1 | 1 | b0 | 0.1 | 0.01 | 6.97 | 0 | 0.07 | 0.12 | 0.1 | 0.09 |
| Cai | ~1 |  | 1 | 1 |  | 0.04 | 0.01 | 2.86 | 0 | 0.01 | 0.07 | 0.04 | 0.04 |
| FDis | ~1 |  | 1 | 1 |  | 0.11 | 0.01 | 7.98 | 0 | 0.08 | 0.14 | 0.11 | 0.11 |
| CWM | ~1 |  | 1 | 1 |  | 0.04 | 0.01 | 3.18 | 0 | 0.02 | 0.06 | 0.04 | 0.04 |
| VPD | ~1 |  | 1 | 1 |  | -0.23 | 0.01 | -17.36 | 0 | -0.26 | -0.2 | -0.23 | -0.24 |
| Cai | ~~ | Cai | 1 | 1 |  | 0.92 | 0.02 | 52.87 | 0 | 0.89 | 0.95 | 0.92 | 0.94 |
| CWM | ~~ | CWM | 1 | 1 |  | 0.81 | 0.01 | 59.38 | 0 | 0.79 | 0.84 | 0.81 | 0.81 |
| FDis | ~~ | FDis | 1 | 1 |  | 0.99 | 0.01 | 66.93 | 0 | 0.96 | 1.02 | 0.99 | 0.99 |
| VPD | ~~ | VPD | 1 | 1 |  | 0.91 | 0.01 | 64.61 | 0 | 0.89 | 0.94 | 0.91 | 1 |
| Cai | ~ | VPD | 2 | 2 | c1 | -0.39 | 0.04 | -8.85 | 0 | -0.48 | -0.3 | -0.39 | -0.25 |
| Cai | ~ | CWM | 2 | 2 | d0 | -0.13 | 0.01 | -9.2 | 0 | -0.16 | -0.1 | -0.13 | -0.13 |
| Cai | ~ | FDis | 2 | 2 | e1 | 0.17 | 0.03 | 6.36 | 0 | 0.12 | 0.23 | 0.17 | 0.17 |
| CWM | ~ | VPD | 2 | 2 | a1 | 0.19 | 0.04 | 4.17 | 0 | 0.1 | 0.28 | 0.19 | 0.12 |
| FDis | ~ | VPD | 2 | 2 | b1 | -0.09 | 0.04 | -2.16 | 0.03 | -0.18 | -0.01 | -0.09 | -0.06 |
| Cai | ~1 |  | 2 | 2 |  | 0.09 | 0.05 | 1.84 | 0.07 | -0.01 | 0.19 | 0.09 | 0.09 |
| FDis | ~1 |  | 2 | 2 |  | -0.29 | 0.05 | -6.16 | 0 | -0.38 | -0.19 | -0.29 | -0.31 |
| CWM | ~1 |  | 2 | 2 |  | 0.1 | 0.05 | 2.17 | 0.03 | 0.01 | 0.2 | 0.1 | 0.11 |
| VPD | ~1 |  | 2 | 2 |  | 0.85 | 0.02 | 50.63 | 0 | 0.82 | 0.89 | 0.85 | 1.35 |
| Cai | ~~ | Cai | 2 | 2 |  | 0.83 | 0.03 | 24.65 | 0 | 0.77 | 0.9 | 0.83 | 0.88 |
| CWM | ~~ | CWM | 2 | 2 |  | 0.9 | 0.03 | 34.7 | 0 | 0.85 | 0.95 | 0.9 | 0.98 |
| FDis | ~~ | FDis | 2 | 2 |  | 0.86 | 0.03 | 26.03 | 0 | 0.79 | 0.92 | 0.86 | 1 |
| VPD | ~~ | VPD | 2 | 2 |  | 0.4 | 0.02 | 26.17 | 0 | 0.37 | 0.43 | 0.4 | 1 |
| b0e0 | := | b0*e0 | 0 | 0 | b0e0 | 0.01 | 0 | 3.67 | 0 | 0 | 0.01 | 0.01 | 0.01 |
| a0d0 | := | a0*d0 | 0 | 0 | a0d0 | -0.06 | 0.01 | -8.8 | 0 | -0.07 | -0.05 | -0.06 | -0.06 |
| total0 | := | c0+(b0*e0)+(a0*d0) | 0 | 0 | total0 | -0.21 | 0.01 | -14.36 | 0 | -0.24 | -0.18 | -0.21 | -0.2 |
| b1e1 | := | b1*e1 | 0 | 0 | b1e1 | -0.02 | 0.01 | -2.05 | 0.04 | -0.03 | 0 | -0.02 | -0.01 |
| a1d1 | := | a1*d1 | 0 | 0 | a1d0 | -0.02 | 0.01 | -3.78 | 0 | -0.04 | -0.01 | -0.02 | -0.02 |
| total1 | := | c1+(b1*e1)+(a1*d1) | 0 | 0 | total1 | -0.43 | 0.04 | -9.7 | 0 | -0.52 | -0.34 | -0.43 | -0.28 |

**Table S13**. Parameter estimates of the parly constrained multigroup structural equation model (Xylem construct) showed in Fig. S8. The paths VPD˜ Cai and VPD˜Fdis were constrained to be invariant between groups. lhs, left-hand-side; op, operators: means is regressed onto; means is corelated with; ˜1 means intercept; rhs, right-hand-side; est, estimated model parameters; se, standard errors for the free parameters in the model; z, value of the estimate divided by its standard error (so-called z-statistic); ci.lower and ci.upper, lower 2.5% and upper 97.5% confidence intervals of z-statistic; p, p values corresponding to the z-statistic evaluated under a standard normal distribution; std, standardized model estimates.

| **lhs** | **op** | **rhs** | **block** | **group** | **label** | **est** | **se** | **z** | **pvalue** | **ci.lower** | **ci.upper** | **std.lv** | **std.all** |
| --- | --- | --- | --- | --- | --- | --- | --- | --- | --- | --- | --- | --- | --- |
| Cai | ~ | VPD | 1 | 1 | c0 | -0.24 | 0.02 | -16.23 | 0 | -0.27 | -0.21 | -0.24 | -0.24 |
| Cai | ~ | CWM | 1 | 1 | d0 | 0.16 | 0.02 | 7.12 | 0 | 0.11 | 0.2 | 0.16 | 0.12 |
| Cai | ~ | FDis | 1 | 1 | e0 | 0.05 | 0.01 | 4.53 | 0 | 0.03 | 0.08 | 0.05 | 0.05 |
| CWM | ~ | VPD | 1 | 1 | a0 | 0.14 | 0.01 | 11.74 | 0 | 0.12 | 0.17 | 0.14 | 0.17 |
| FDis | ~ | VPD | 1 | 1 | b0 | 0.2 | 0.01 | 15.24 | 0 | 0.17 | 0.22 | 0.2 | 0.21 |
| Cai | ~1 |  | 1 | 1 |  | 0.03 | 0.01 | 1.88 | 0.06 | 0 | 0.05 | 0.03 | 0.03 |
| FDis | ~1 |  | 1 | 1 |  | 0.05 | 0.01 | 3.35 | 0 | 0.02 | 0.07 | 0.05 | 0.05 |
| CWM | ~1 |  | 1 | 1 |  | 0.1 | 0.01 | 7.7 | 0 | 0.07 | 0.12 | 0.1 | 0.12 |
| VPD | ~1 |  | 1 | 1 |  | -0.23 | 0.01 | -17.36 | 0 | -0.26 | -0.2 | -0.23 | -0.24 |
| Cai | ~~ | Cai | 1 | 1 |  | 0.92 | 0.02 | 52.65 | 0 | 0.88 | 0.95 | 0.92 | 0.94 |
| CWM | ~~ | CWM | 1 | 1 |  | 0.61 | 0.08 | 7.33 | 0 | 0.45 | 0.77 | 0.61 | 0.97 |
| FDis | ~~ | FDis | 1 | 1 |  | 0.79 | 0.03 | 29.74 | 0 | 0.74 | 0.84 | 0.79 | 0.96 |
| VPD | ~~ | VPD | 1 | 1 |  | 0.91 | 0.01 | 64.61 | 0 | 0.89 | 0.94 | 0.91 | 1 |
| Cai | ~ | VPD | 2 | 2 | c1 | -0.4 | 0.05 | -8.69 | 0 | -0.49 | -0.31 | -0.4 | -0.26 |
| Cai | ~ | CWM | 2 | 2 | d1 | 0.1 | 0.02 | 5.15 | 0 | 0.06 | 0.13 | 0.1 | 0.15 |
| Cai | ~ | FDis | 2 | 2 | e0 | 0.05 | 0.01 | 4.53 | 0 | 0.03 | 0.08 | 0.05 | 0.07 |
| CWM | ~ | VPD | 2 | 2 | a1 | -0.47 | 0.06 | -7.47 | 0 | -0.6 | -0.35 | -0.47 | -0.19 |
| FDis | ~ | VPD | 2 | 2 | b0 | 0.2 | 0.01 | 15.24 | 0 | 0.17 | 0.22 | 0.2 | 0.1 |
| Cai | ~1 |  | 2 | 2 |  | 0.03 | 0.05 | 0.54 | 0.59 | -0.07 | 0.13 | 0.03 | 0.03 |
| FDis | ~1 |  | 2 | 2 |  | -0.17 | 0.04 | -4.62 | 0 | -0.25 | -0.1 | -0.17 | -0.13 |
| CWM | ~1 |  | 2 | 2 |  | 0.13 | 0.05 | 2.79 | 0.01 | 0.04 | 0.22 | 0.13 | 0.08 |
| VPD | ~1 |  | 2 | 2 |  | 0.85 | 0.02 | 50.63 | 0 | 0.82 | 0.89 | 0.85 | 1.35 |
| Cai | ~~ | Cai | 2 | 2 |  | 0.86 | 0.03 | 24.7 | 0 | 0.79 | 0.93 | 0.86 | 0.89 |
| CWM | ~~ | CWM | 2 | 2 |  | 2.35 | 0.42 | 5.59 | 0 | 1.52 | 3.17 | 2.35 | 0.96 |
| FDis | ~~ | FDis | 2 | 2 |  | 1.69 | 0.11 | 15.65 | 0 | 1.48 | 1.9 | 1.69 | 0.99 |
| VPD | ~~ | VPD | 2 | 2 |  | 0.4 | 0.02 | 26.17 | 0 | 0.37 | 0.43 | 0.4 | 1 |
| b0e0 | := | b0*e0 | 0 | 0 | b0e0 | 0.01 | 0 | 4.33 | 0 | 0.01 | 0.02 | 0.01 | 0.01 |
| a0d0 | := | a0*d0 | 0 | 0 | a0d0 | 0.02 | 0 | 5.24 | 0 | 0.01 | 0.03 | 0.02 | 0.02 |
| total0 | := | c0+(b0*e0)+(a0*d0) | 0 | 0 | total0 | -0.21 | 0.01 | -14.27 | 0 | -0.24 | -0.18 | -0.21 | -0.2 |
| b1e1 | := | b0*e1 | 0 | 0 | b0e0 | 0.01 | 0 | 4.33 | 0 | 0.01 | 0.02 | 0.01 | 0.01 |
| a1d0 | := | a1*d1 | 0 | 0 | a1d1 | -0.05 | 0.01 | -5.35 | 0 | -0.06 | -0.03 | -0.05 | -0.03 |
| total1 | := | c0+(b0*e1)+(a1*d1) | 0 | 0 | total1 | -0.43 | 0.04 | -9.66 | 0 | -0.52 | -0.34 | -0.43 | -0.28 |

**Table S14**. Parameter estimates of the partly constrained multigroup structural equation model (Dim1 construct) showed in Fig. S9.The paths VPD˜ Cai and CWM˜ Cai were constrained to be invariant between groups. lhs, left-hand-side; op, operators: means is regressed onto; means is corelated with; ˜1 means intercept; rhs, right-hand-side; est, estimated model parameters; se, standard errors for the free parameters in the model; z, value of the estimate divided by its standard error (so-called z-statistic); ci.lower and ci.upper, lower 2.5% and upper 97.5% confidence intervals of z-statistic; p, p values corresponding to the z-statistic evaluated under a standard normal distribution; std, standardized model estimates.

| **lhs** | **op** | **rhs** | **block** | **group** | **label** | **est** | **se** | **z** | **pvalue** | **ci.lower** | **ci.upper** | **std.lv** | **std.all** |
| --- | --- | --- | --- | --- | --- | --- | --- | --- | --- | --- | --- | --- | --- |
| Cai | ~ | VPD | 1 | 1 | c0 | -0.21 | 0.02 | -13.89 | 0 | -0.24 | -0.18 | -0.21 | -0.2 |
| Cai | ~ | CWM | 1 | 1 | d0 | -0.02 | 0.01 | -1.79 | 0.07 | -0.05 | 0 | -0.02 | -0.02 |
| Cai | ~ | FDis | 1 | 1 | e0 | 0.03 | 0.01 | 2.21 | 0.03 | 0 | 0.06 | 0.03 | 0.03 |
| CWM | ~ | VPD | 1 | 1 | a0 | 0.22 | 0.01 | 18.31 | 0 | 0.2 | 0.25 | 0.22 | 0.23 |
| FDis | ~ | VPD | 1 | 1 | b0 | 0.11 | 0.01 | 8.19 | 0 | 0.09 | 0.14 | 0.11 | 0.11 |
| Cai | ~1 |  | 1 | 1 |  | 0.04 | 0.01 | 2.68 | 0.01 | 0.01 | 0.07 | 0.04 | 0.04 |
| FDis | ~1 |  | 1 | 1 |  | 0.12 | 0.01 | 8.52 | 0 | 0.09 | 0.14 | 0.12 | 0.12 |
| CWM | ~1 |  | 1 | 1 |  | -0.02 | 0.01 | -1.49 | 0.14 | -0.05 | 0.01 | -0.02 | -0.02 |
| VPD | ~1 |  | 1 | 1 |  | -0.23 | 0.01 | -17.36 | 0 | -0.26 | -0.2 | -0.23 | -0.24 |
| Cai | ~~ | Cai | 1 | 1 |  | 0.93 | 0.02 | 52.71 | 0 | 0.9 | 0.97 | 0.93 | 0.96 |
| CWM | ~~ | CWM | 1 | 1 |  | 0.85 | 0.02 | 47.46 | 0 | 0.82 | 0.89 | 0.85 | 0.95 |
| FDis | ~~ | FDis | 1 | 1 |  | 0.95 | 0.01 | 72.57 | 0 | 0.93 | 0.98 | 0.95 | 0.99 |
| VPD | ~~ | VPD | 1 | 1 |  | 0.91 | 0.01 | 64.61 | 0 | 0.89 | 0.94 | 0.91 | 1 |
| Cai | ~ | VPD | 2 | 2 | c1 | -0.43 | 0.04 | -9.65 | 0 | -0.52 | -0.34 | -0.43 | -0.28 |
| Cai | ~ | CWM | 2 | 2 | d0 | -0.02 | 0.01 | -1.79 | 0.07 | -0.05 | 0 | -0.02 | -0.03 |
| Cai | ~ | FDis | 2 | 2 | e1 | 0.11 | 0.03 | 4.15 | 0 | 0.06 | 0.16 | 0.11 | 0.11 |
| CWM | ~ | VPD | 2 | 2 | a1 | 0.05 | 0.06 | 0.81 | 0.42 | -0.07 | 0.17 | 0.05 | 0.03 |
| FDis | ~ | VPD | 2 | 2 | b1 | 0 | 0.05 | -0.03 | 0.98 | -0.09 | 0.09 | 0 | 0 |
| Cai | ~1 |  | 2 | 2 |  | 0.08 | 0.05 | 1.48 | 0.14 | -0.02 | 0.17 | 0.08 | 0.08 |
| FDis | ~1 |  | 2 | 2 |  | -0.36 | 0.05 | -7.69 | 0 | -0.46 | -0.27 | -0.36 | -0.37 |
| CWM | ~1 |  | 2 | 2 |  | 0.29 | 0.06 | 4.51 | 0 | 0.16 | 0.41 | 0.29 | 0.25 |
| VPD | ~1 |  | 2 | 2 |  | 0.85 | 0.02 | 50.63 | 0 | 0.82 | 0.89 | 0.85 | 1.35 |
| Cai | ~~ | Cai | 2 | 2 |  | 0.86 | 0.03 | 24.72 | 0 | 0.8 | 0.93 | 0.86 | 0.91 |
| CWM | ~~ | CWM | 2 | 2 |  | 1.33 | 0.05 | 25.17 | 0 | 1.23 | 1.43 | 1.33 | 1 |
| FDis | ~~ | FDis | 2 | 2 |  | 0.97 | 0.03 | 30.32 | 0 | 0.91 | 1.04 | 0.97 | 1 |
| VPD | ~~ | VPD | 2 | 2 |  | 0.4 | 0.02 | 26.17 | 0 | 0.37 | 0.43 | 0.4 | 1 |
| b0e0 | := | b0*e0 | 0 | 0 | b0e0 | 0 | 0 | 2.14 | 0.03 | 0 | 0.01 | 0 | 0 |
| a0d0 | := | a0*d0 | 0 | 0 | a0d0 | -0.01 | 0 | -1.77 | 0.08 | -0.01 | 0 | -0.01 | -0.01 |
| total0 | := | c0+(b0*e0)+(a0*d0) | 0 | 0 | total0 | -0.21 | 0.01 | -14.35 | 0 | -0.24 | -0.18 | -0.21 | -0.21 |
| b1e1 | := | b1*e1 | 0 | 0 | b1e1 | 0 | 0.01 | -0.03 | 0.98 | -0.01 | 0.01 | 0 | 0 |
| a1d0 | := | a1*d0 | 0 | 0 | a1d0 | 0 | 0 | -0.73 | 0.46 | 0 | 0 | 0 | 0 |
| total1 | := | c0+(b1*e1)+(a1*d0) | 0 | 0 | total1 | -0.43 | 0.04 | -9.67 | 0 | -0.52 | -0.34 | -0.43 | -0.28 |
|  |  |  |  |  |  |  |  |  |  |  |  |  |  |

**Supplementary Figures**

**Fig S1.** Left panel shows the geographical distribution of NFI forest plots over imposed to vapor pressure deficit (VPD, in kPA) raster map considered in our study (Abatzoglou et al. 2018). Theoretical model (inset) of climate (VPD) and community functional properties (CWM and FDis) on annual volume increment (Prod). Solid arrows indicate predicted causal relationships among variables and lowercase letters are path estimates. On right panel the temperate and Mediterranean bioclimatic domains.


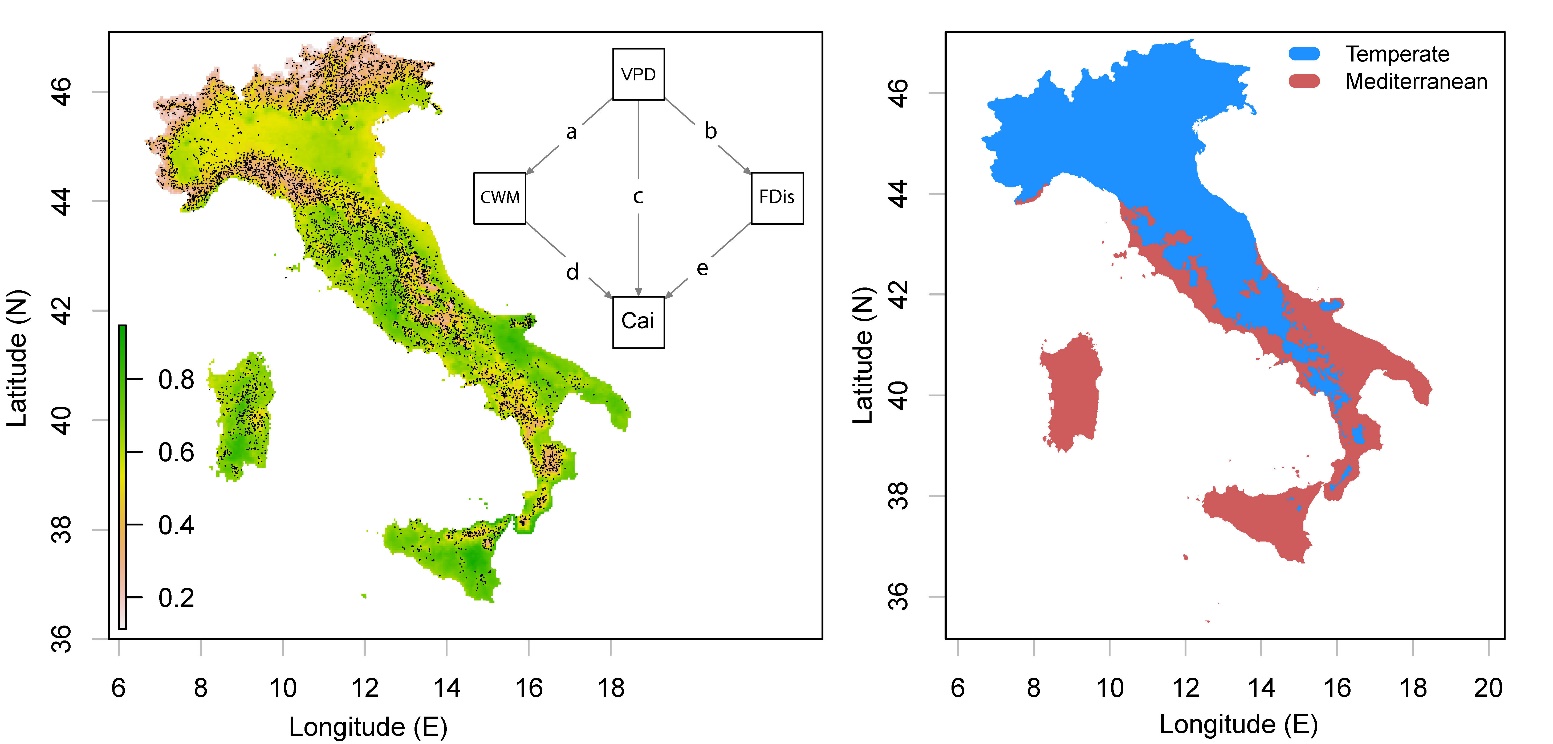


**Fig S2.** Principal Component Analysis: A) Variable correlation plot showing variable relationships (i.e., positively correlated functional traits group together). Colors show the relative contribution of each variable to principal components 1 and 2. B) Positions of species, from the Italian national forest inventory, in PCA space (Oksanen et al. 2019, Kassambara et al. 2020).


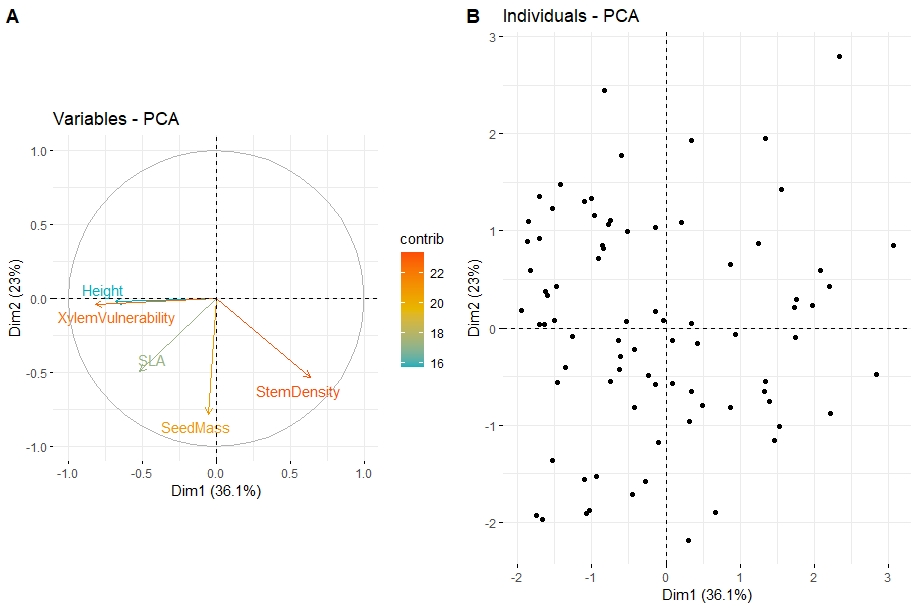


**Fig S3.** Relationship between species richness (SpRich) and functional dispersion (FDis) for seed mass (FDis_SeedMAss_, r(6503) = 0.15, p <0.001), tree height (FDis_Height_, r(6499) = 0.08, p <0.001), specific leaf area (FDis_SLA_, r(6481) = 0.13, p <0.001), wood density (FDis_WD_, r(6539) = 0.15, p <0.001), xylem vulnerability (FDis_Xylem_, r(6385) = 0.04, p <0.001) function traits, and the functional dispersion of functional dispersion all traits (FDis_All_, r(6498) = 0.14, p <0.001). Straight lines represent predicted linear regression.


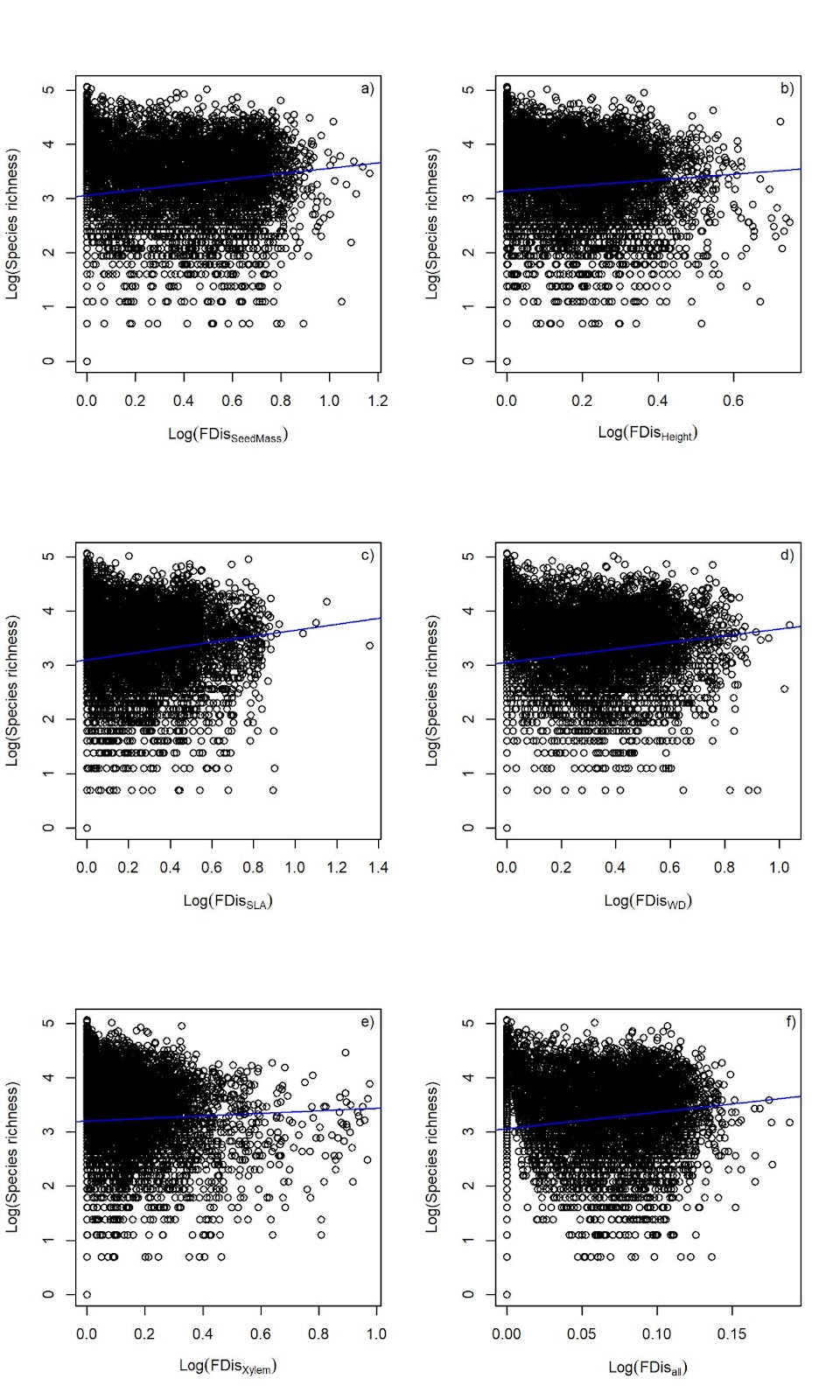


**Fig S4.** Results of multi group structural equation modeling (MGSEM), with seed mass as the functional trait, for the temperate (a) and Mediterranean (b) bioclimatic domains. Arrowheads lines represent causal paths and bidirectional arrowhead indicates residual variance, with superimposed standardized partial regression coefficients. The significance *p* of paths is reported in Tab. S10. Squares represent manifest variables. At the top, model's fit indexes: CFI, comparative fit index; TLI, Tucker–Lewis index; RMSEA, root mean square error of approximation index; SRMR, standardized root mean square residual.

**
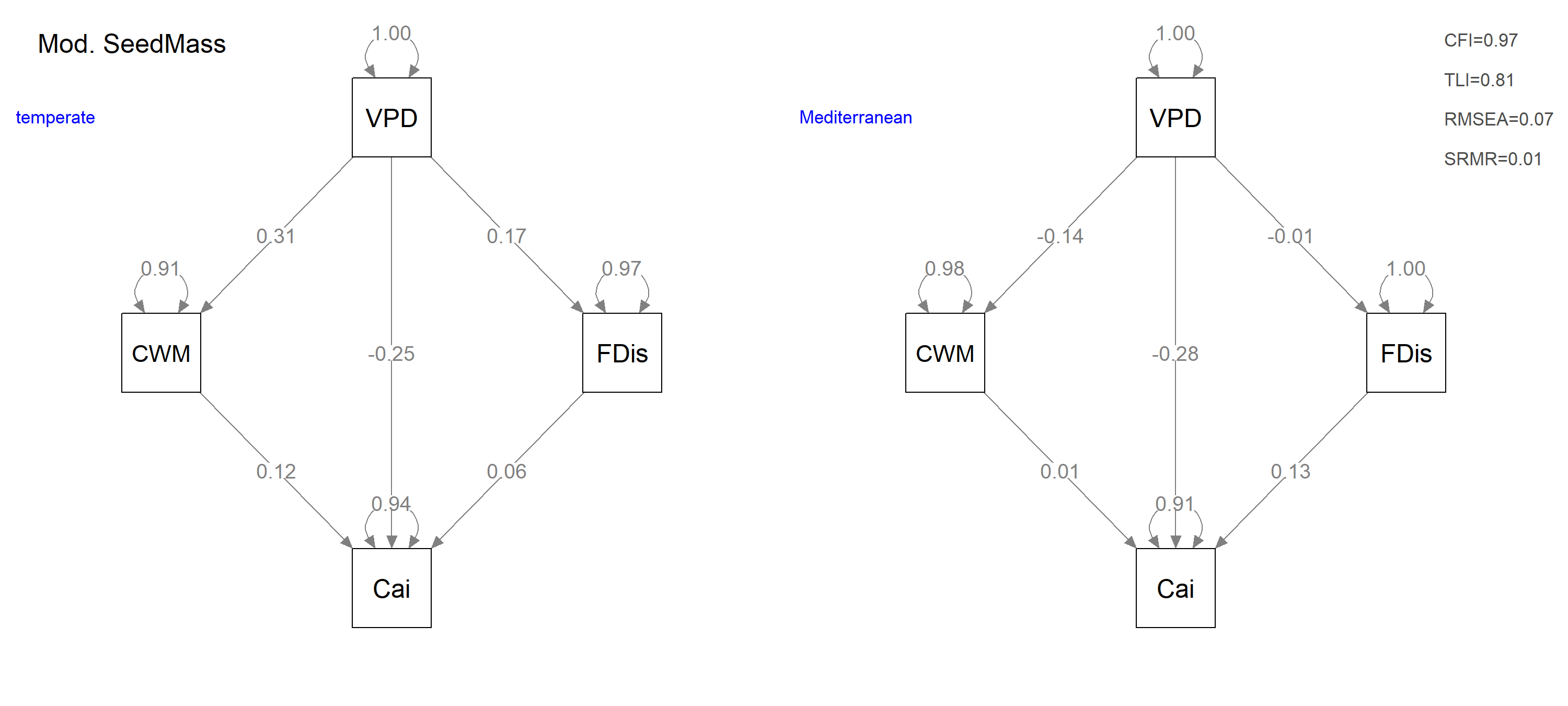
**

**Fig S5.** Results of multi group structural equation modeling (MGSEM), with height as the functional trait, for the temperate (a) and Mediterranean (b) bioclimatic domains. Arrowheads lines represent causal paths and bidirectional arrowhead indicates residual variance, with superimposed standardized partial regression coefficients. The significance *p* of paths is reported in Tab. S11. Squares represent manifest variables. At the top, model's fit indexes: CFI, comparative fit index; TLI, Tucker–Lewis index; RMSEA, root mean square error of approximation index; SRMR, standardized root mean square residual.


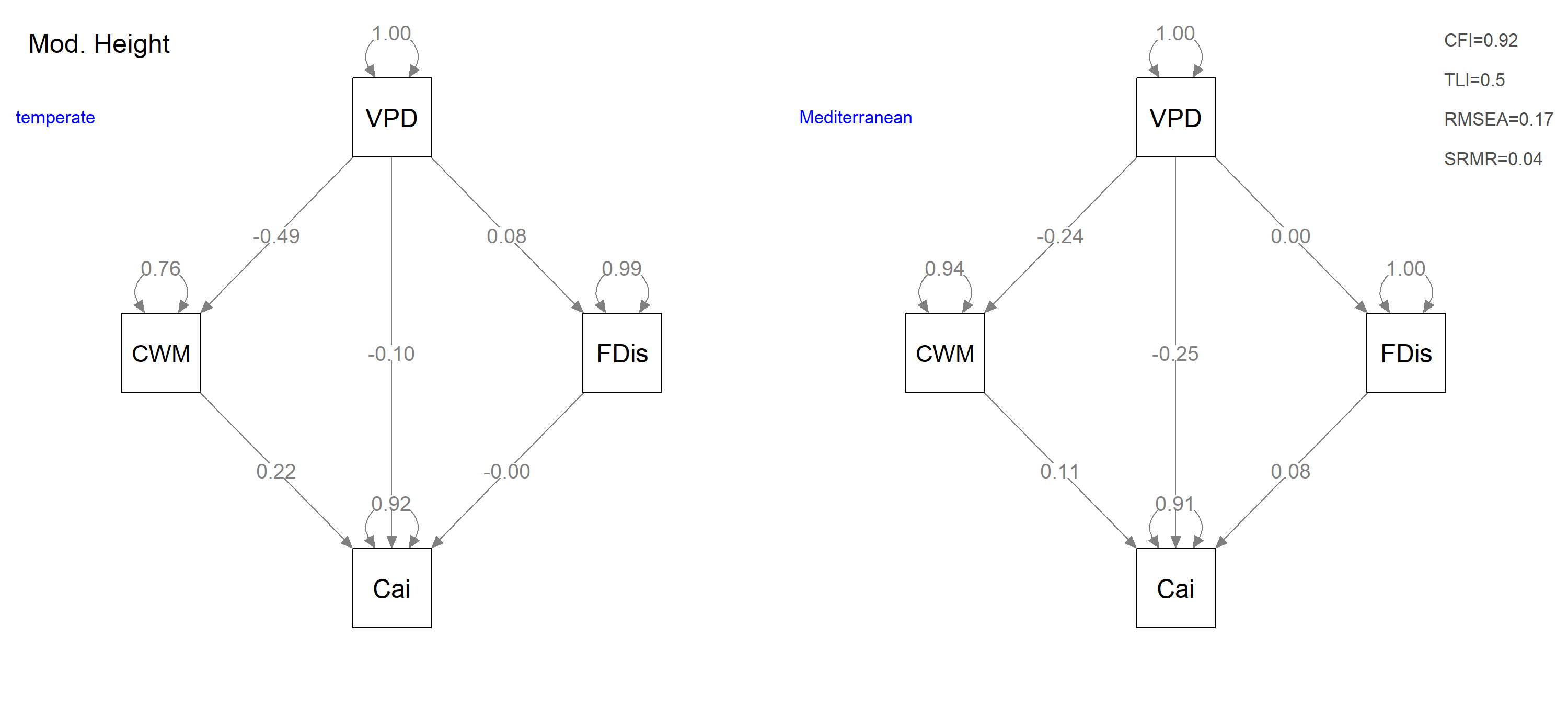


**Fig S6.** Results of multi group structural equation modeling (MGSEM), with SLA as the functional trait, for the temperate (a) and Mediterranean (b) bioclimatic domains. Arrowheads lines represent causal paths and bidirectional arrowhead indicates residual variance, with superimposed standardized partial regression coefficients. The significance *p* of paths is reported in Tab. S12. Squares represent manifest variables. At the top, model's fit indexes: CFI, comparative fit index; TLI, Tucker–Lewis index; RMSEA, root mean square error of approximation index; SRMR, standardized root mean square residual.


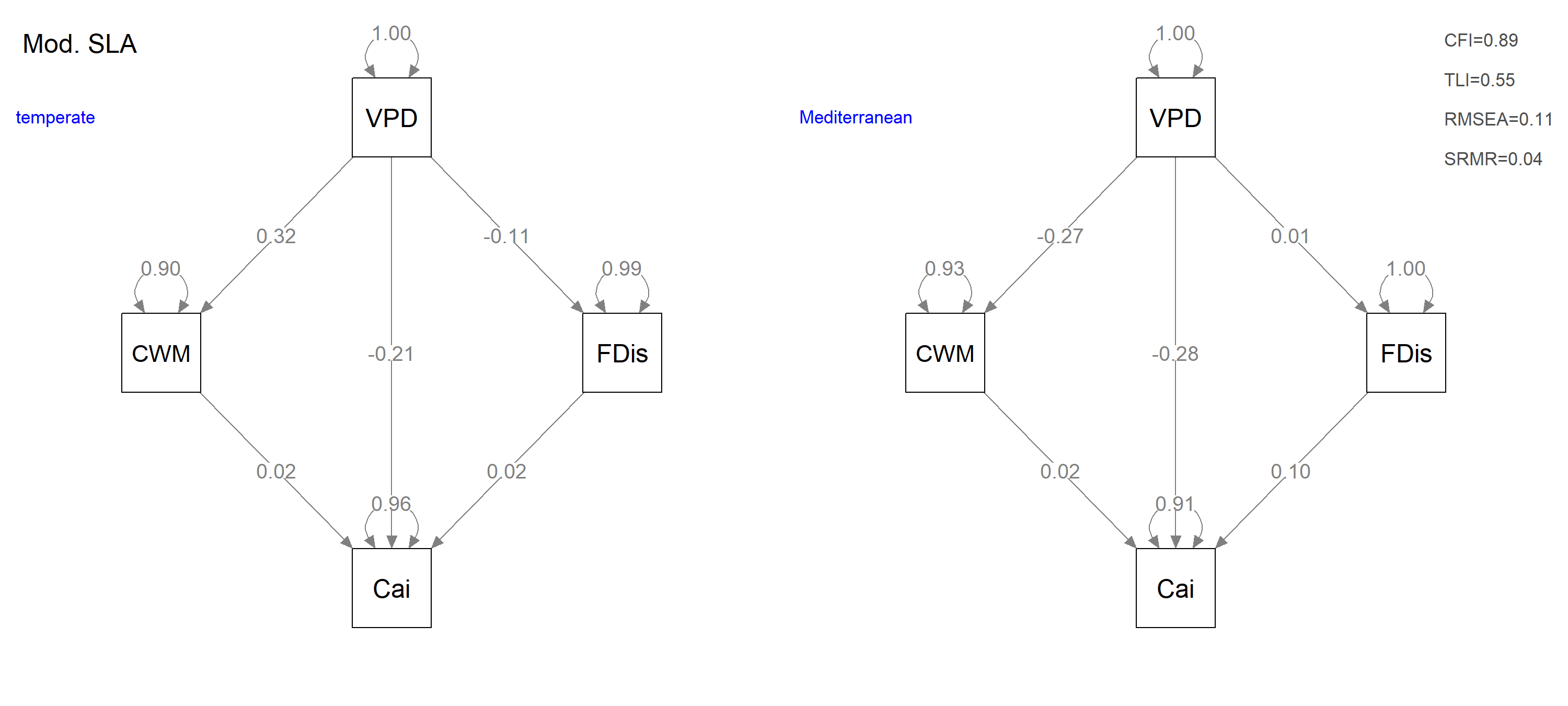


**Fig S7.** Results of multi group structural equation modeling (MGSEM), with wood density as the functional trait, for the temperate (a) and Mediterranean (b) bioclimatic domains. Arrowheads lines represent causal paths and bidirectional arrowhead indicates residual variance, with superimposed standardized partial regression coefficients. The significance *p* of paths is reported in Tab. S13. Squares represent manifest variables. At the top, model's fit indexes: CFI, comparative fit index; TLI, Tucker–Lewis index; RMSEA, root mean square error of approximation index; SRMR, standardized root mean square residual.


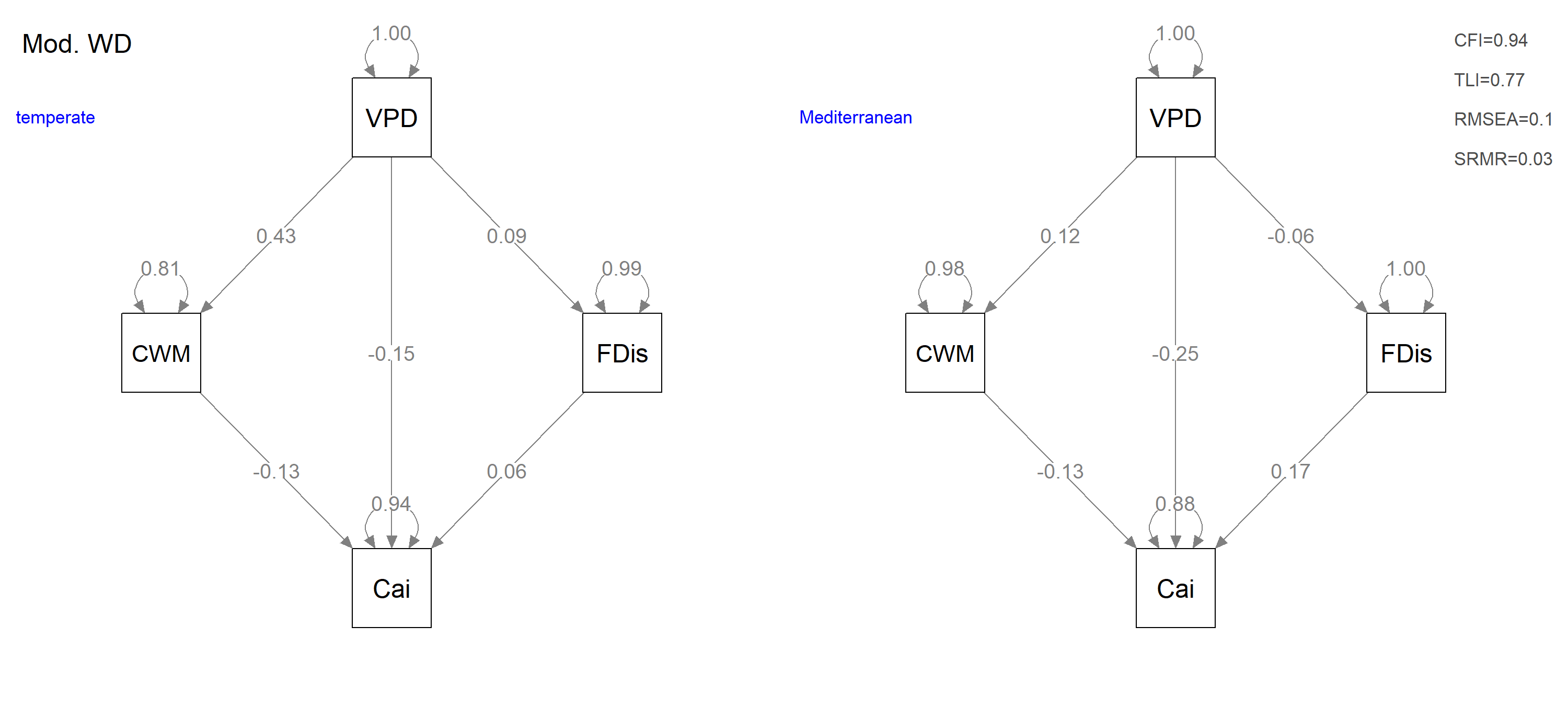


**Fig S8.** Results of multi group structural equation modeling (MGSEM), with xylem vulnerability (P50) as the functional trait, for the temperate (a) and Mediterranean (b) bioclimatic domains. Arrowheads lines represent causal paths and bidirectional arrowhead indicates residual variance, with superimposed standardized partial regression coefficients. The significance *p* of paths is reported in Tab. S14. Squares represent manifest variables. At the top, model's fit indexes: CFI, comparative fit index; TLI, Tucker–Lewis index; RMSEA, root mean square error of approximation index; SRMR, standardized root mean square residual.


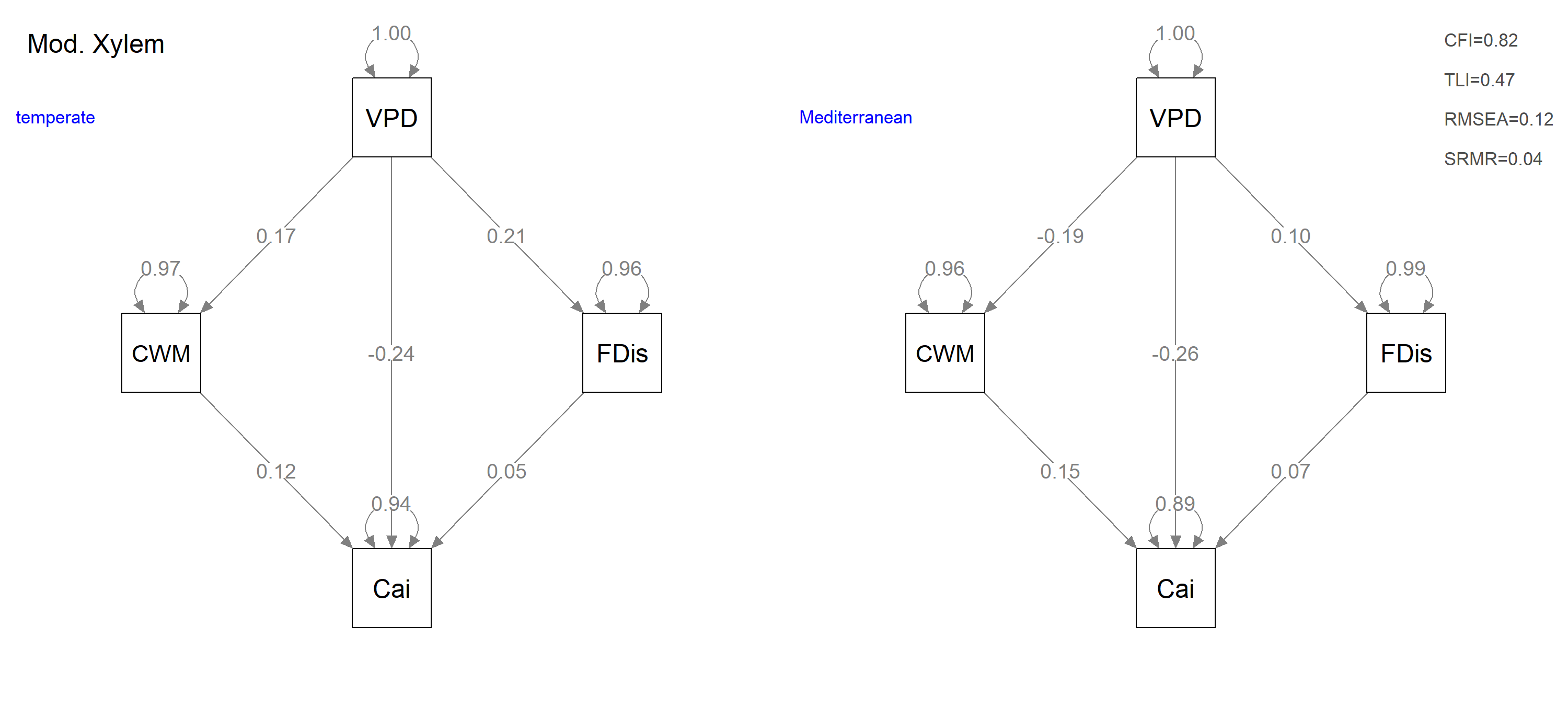


**Fig S9.** Results of multi group structural equation modeling (MGSEM), with suit of traits as the functional trait, for the temperate (a) and Mediterranean (b) bioclimatic domains. Arrowheads lines represent causal paths and bidirectional arrowhead indicates residual variance, with superimposed standardized partial regression coefficients. The significance *p* of paths is reported in Tab. S15. Squares represent manifest variables. At the top, model's fit indexes: CFI, comparative fit index; TLI, Tucker–Lewis index; RMSEA, root mean square error of approximation index; SRMR, standardized root mean square residual.


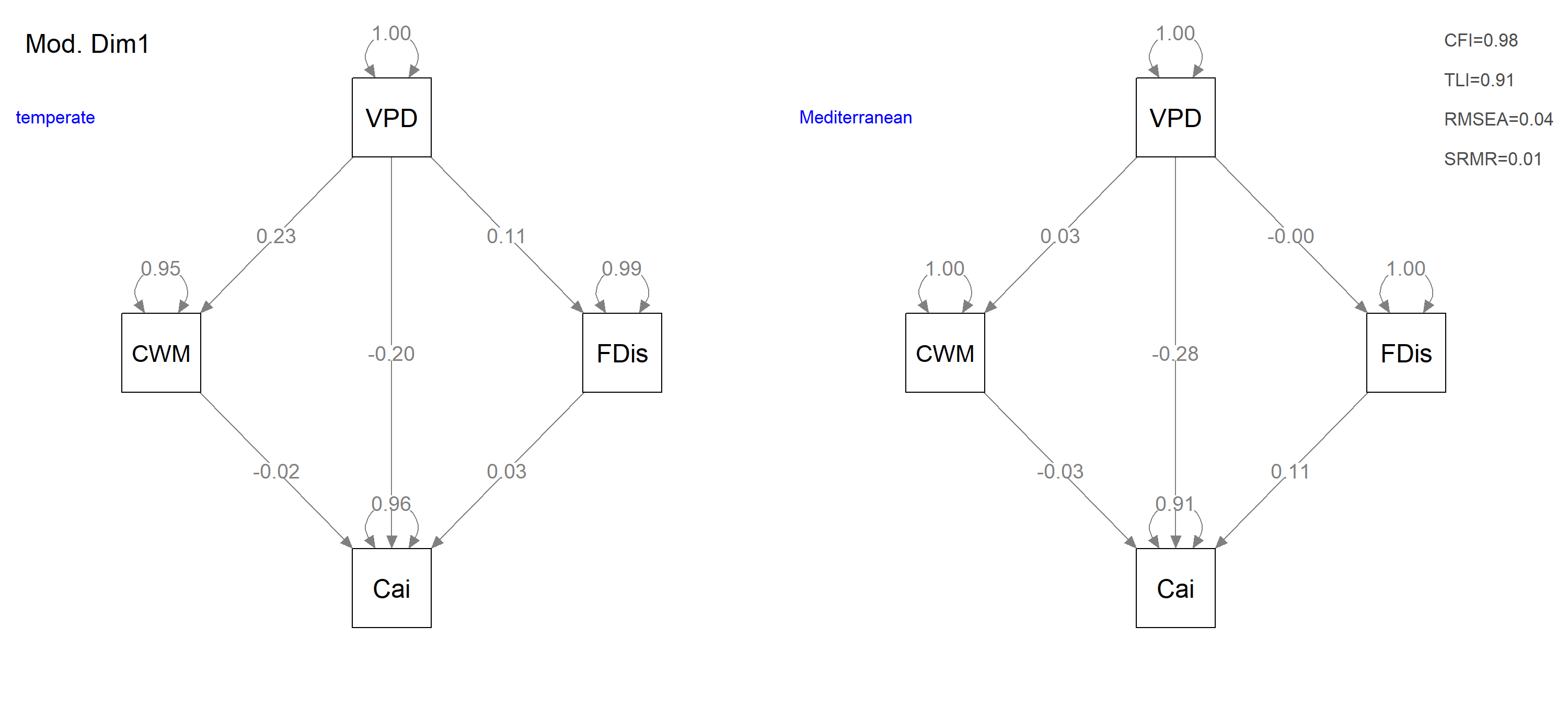


**Fig S10.** Relationship between community weighted mean of proportion of angiosperm and vapor pressure deficit (VPD) for forest plots within the Temperate climatic region. Green line represents predicted logistic regression.

**
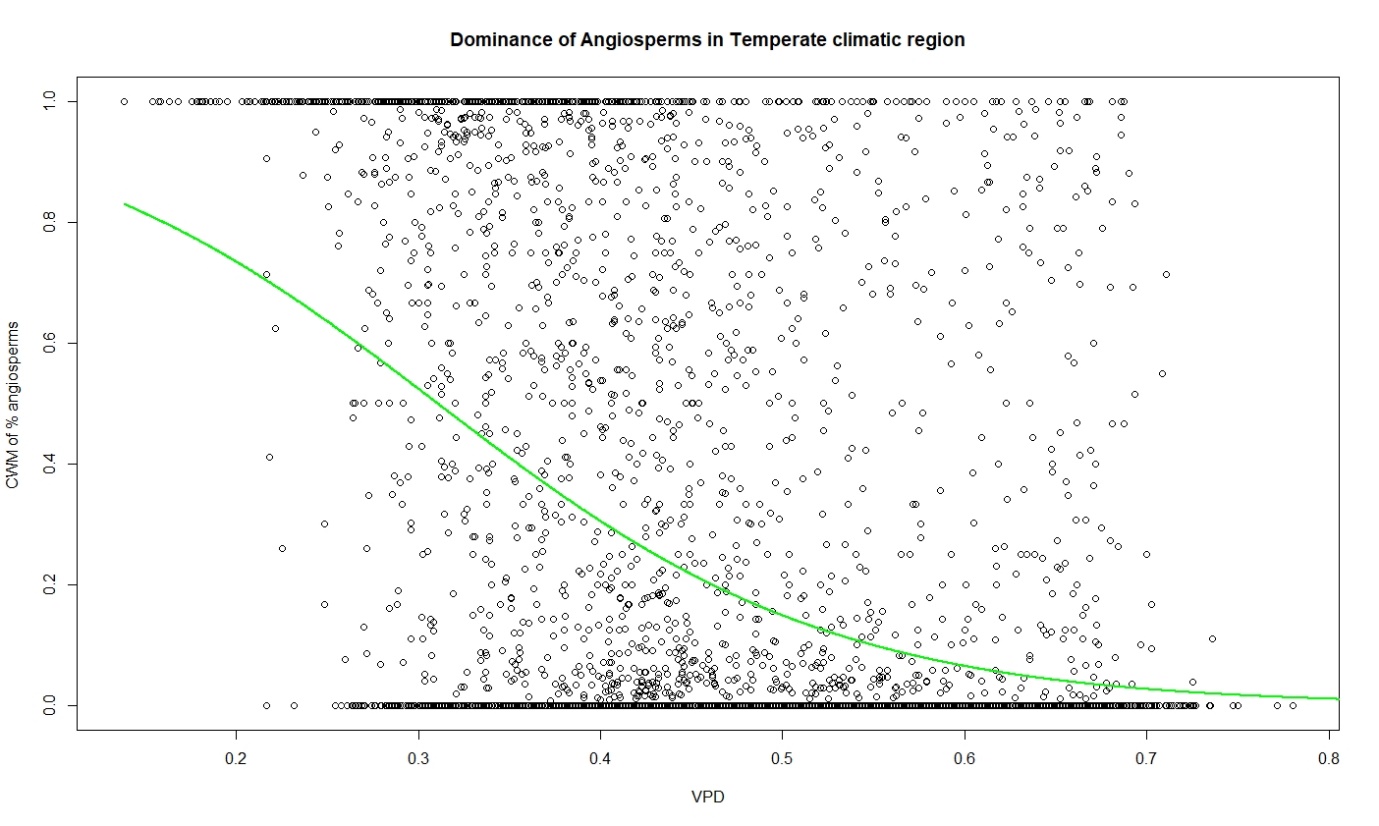
**

**Fig S11.** Histograms for parameters used in structural equation modeling.


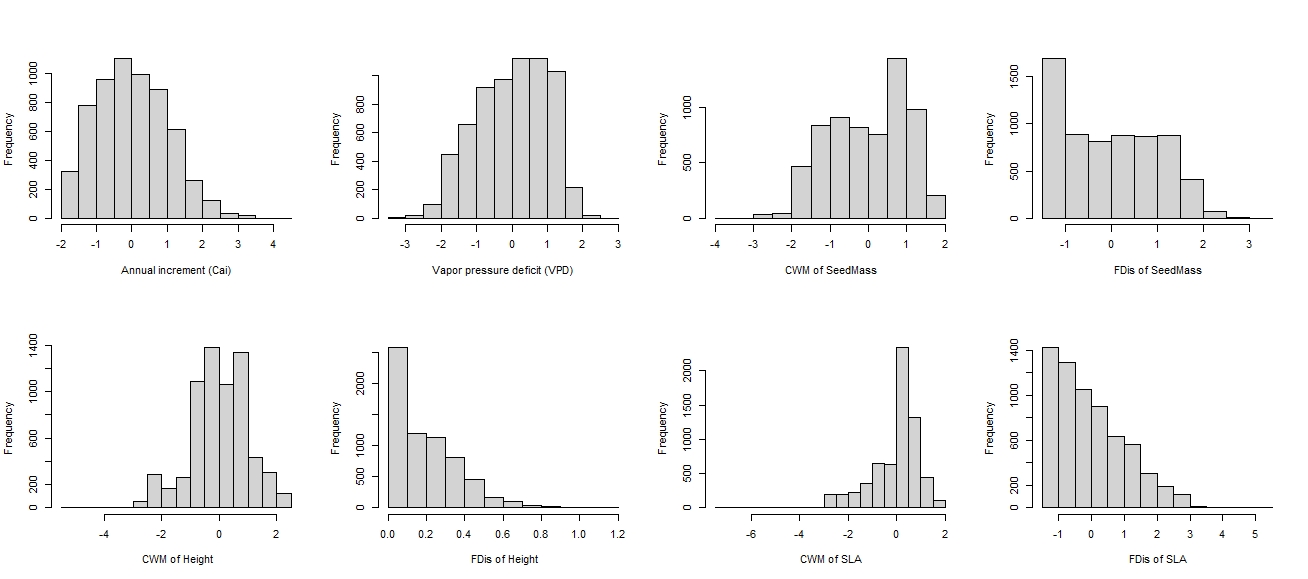


**Fig S12.** Histograms for parameters used in structural equation modeling.


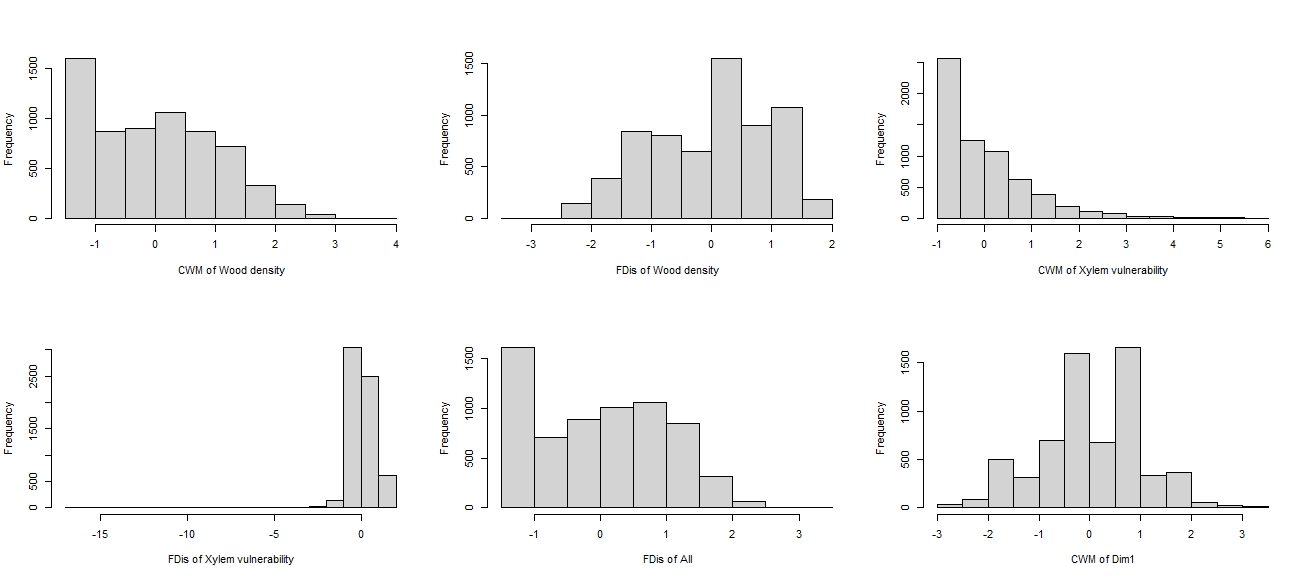

Supplement: Supplementary file 1 — Appendix S1. [file ECE3-13-e10406-s001.docx]
